# Co-application of canavanine and irradiation uncouples anticancer potential of arginine deprivation from citrulline availability

**DOI:** 10.18632/oncotarget.12320

**Published:** 2016-09-28

**Authors:** Yuliya Kurlishchuk, Bozhena Vynnytska-Myronovska, Philipp Grosse-Gehling, Yaroslav Bobak, Friederike Manig, Oleg Chen, Sebastian R. Merker, Thomas Henle, Steffen Löck, Daniel E. Stange, Oleh Stasyk, Leoni A. Kunz

**Affiliations:** ^1^ OncoRay–National Center for Radiation Research in Oncology, Faculty of Medicine and University Hospital Carl Gustav Carus, TU Dresden and Helmholtz-Zentrum Dresden-Rossendorf, Institute of Radiooncology, Dresden, Germany; ^2^ Department of Cell Signaling, Institute of Cell Biology, National Academy of Sciences of Ukraine, Lviv, Ukraine; ^3^ Institute of Food Chemistry, TU Dresden, Dresden, Germany; ^4^ Department of Gastrointestinal, Thoracic and Vascular Surgery, University Hospital Carl Gustav Carus, TU Dresden, Dresden, Germany; ^5^ Department of Oncology, University of Oxford, Old Road Campus Research Building, Oxford, UK; ^6^ Current address: Clinic of Urology and Pediatric Urology, Saarland University Medical Center, Homburg/Saar, Germany

**Keywords:** arginine deprivation, ASS1 expression, colorectal cancer (CRC), CRC spheroids, normal colon organoids

## Abstract

The moderate anticancer effect of arginine deprivation in clinical trials has been linked to an induced argininosuccinate synthetase (ASS1) expression in initially ASS1-negative tumors, and ASS1-positive cancers are anticipated as non-responders. Our previous studies indicated that arginine deprivation and low doses of the natural arginine analog canavanine can enhance radioresponse. However, the efficacy of the proposed combination in the presence of extracellular citrulline, the substrate for arginine synthesis by ASS1, remains to be elucidated, in particular for malignant cells with positive and/or inducible ASS1 as in colorectal cancer (CRC). Here, the physiological citrulline concentration of 0.05 mM was insufficient to overcome cell cycle arrest and radiosensitization triggered by arginine deficiency. Hyperphysiological citrulline (0.4 mM) did not entirely compensate for the absence of arginine and significantly decelerated cell cycling. Similar levels of canavanine-induced apoptosis were detected in the absence of arginine regardless of citrulline supplementation both in 2-D and advanced 3-D assays, while normal colon epithelial cells in organoid/colonosphere culture were unaffected. Notably, canavanine tremendously enhanced radiosensitivity of arginine-starved 3-D CRC spheroids even in the presence of hyperphysiological citrulline. We conclude that the novel combinatorial targeting strategy of metabolic-chemo-radiotherapy has great potential for the treatment of malignancies with inducible ASS1 expression.

## INTRODUCTION

Arginine is a multifaceted amino acid, which is required not only for protein synthesis, but also for the production of many other cellular metabolites, e.g. urea, nitric oxide, polyamines, proline, glutamate, creatine, and agmatine [1]. The metabolism of this amino acid is a complex process with tissue and organ-specific patterns [2, 3]. In human body under the normal physiological conditions cells meet their arginine needs to varying degree by a direct uptake from the bloodstream and its *de novo* synthesis. The non-proteinogenic amino acid citrulline, which is also supplied via the blood/plasma, is a key arginine precursor and becomes more relevant for cell survival under arginine shortage [2, 4]. Two tightly coupled enzymes are required for the intracellular conversion of citrulline to arginine, i.e. argininosuccinate synthetase (ASS1, EC 6.3.4.5) and argininosuccinate lyase (ASL, EC 4.3.2.1) [1].

Cancer cells have higher nutrient demands than normal non-malignant cells due to their accelerated metabolic and proliferation rates [5]. Some of them become auxotrophic for arginine and depend on the exogenous supply of this amino acid [4, 6]. Critically reduced ASS1 enzyme level can result in the inability of cancer cells to utilize citrulline for arginine synthesis and ASS1 deficiency was thus adopted as a marker of arginine auxotrophy and sensitivity to arginine deprivation [7–9]. Discovery and improvement of arginine-degrading enzymes, such as bacterial arginine deiminase (ADI, EC 3.5.3.6) and recombinant human arginase 1 (rhARG, EC 3.5.3.1), allowed to progress from *in vitro* to *in vivo* experiments [10, 11] and finally to translate the approach into the clinics. By now, the therapeutic potential of arginine deprivation has been established in clinical trials for melanomas and hepatocellular carcinomas [12–15]; trials on other ASS1-deficient malignancies are underway (e.g. leukemia (NCT01910012), lymphoma (NCT01910025), prostate cancer (NCT01497925, NCT02285101) etc., all from (http://clinicaltrials.gov).

Arginine deprivation treatment strategies are not yet considered for tumor entities, which initially have a detectable amount of ASS1 protein or could induce expression of *ASS1* gene upon arginine starvation [4, 6, 7, 16]. Rationale for this is the putative compensatory effect of citrulline-to-arginine conversion in the *in vivo* situation. Amongst others, human colorectal cancer (CRC) falls into the ASS1-positive category due to the high ASS1 protein level detected in the majority of CRC tissue samples in early studies [7, 17]. Consequently, CRC was excluded from the list of tumors defined as the responders to arginine deprivation therapy [4, 7]. However, our recent *in vitro* data indicate that cancer cells might be radiosensitized in the absence of arginine even if they express citrulline-to-arginine converting enzymes [18, 19]. Therefore, it is reasonable to speculate that arginine deprivation therapy could prime CRC and other ASS1-positive malignancies to both standard-of-care and novel combinational therapies. Here we propose to co-apply arginine deprivation with a natural arginine analog canavanine, as such combination supposedly preserves the cytotoxic potential of canavanine [20–22] with a selectively high anticancer efficacy *in vitro* as indicated in an earlier study [23].

The anticipated ability of CRC cells to utilize citrulline for arginine synthesis could be a severe obstacle for arginine deprivation-based treatment regimes. The present study was thus designed to 1) prove that, despite the inducible mode of ASS1 expression, arginine metabolism can still be considered as a promising target in CRC treatment and 2) gain an insight into the presumed adverse compensatory mechanism of citrulline conversion to arginine. Human CRC cell lines were grown both in conventional two-dimensional (2-D) monolayer cultures, as well as in 3-D spheroid cultures, which were proposed as a more reliable tool for evaluating metabolic anticancer therapies before turning into whole animal studies [19].

## RESULTS

### ASS1 protein expression in human CRC cell lines

**Figure 1:**
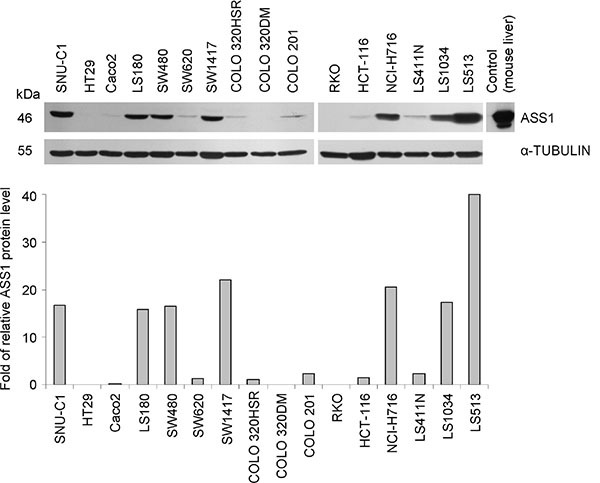
ASS1 expression in human CRC cell lines Representative Western blot and densitometric analysis of protein bands relative to an α-TUBULIN loading control (bottom) show ASS1 levels in CRC cell lines grown in monolayer culture under regular (arginine-rich) conditions.

As CRC has been claimed to be an ASS1-positive tumor entity, we initially monitored ASS1 level in protein extracts from 16 established human CRC cell lines. These cell lines essentially differ in their genetic and epigenetic profiles covering the most frequent alterations related to colorectal carcinogenesis as described in Supplementary Table S1. In regular medium, ASS1 protein expression was high in only 7/16 cell lines while 9/16 showed low or undetectable ASS1 levels in Western blot analysis when grown in monolayer culture (Figure 1). Grouping them into high and low ASS1 expressors (Figure 1, Supplementary Table S1) allowed us comparing the genetic profiles of these two categories. Evidentially, no correlation of basal amount of ASS1 protein with either microsatellite stability or *TP53*, *BRAF* and *APC* gene status could be identified. Only a weak correlation (*p* = 0.049, *R* = 0.49) was seen between ASS1 protein level and mutations in *KRAS* gene. On the contrary, a CpG methylator phenotype in CRC cells seems to be accompanied by reduced ASS1 production rates as indicated by a highly significant negative correlation with ASS1 level (*p* = 0.005, *R* = −0.68). This observation is reasonable, as CpG promoter methylation was shown to cause epigenetic silencing of *ASS1* gene in other tumor entities [9, 24, 25].

In order to verify if ASS1 upregulation upon arginine deprivation is hampered in CRC cells with a CpG methylator phenotype, the genetically most divergent ASS1 low/non-expressor cell lines HCT-116 and HT29, which reflect the two major pathways of CRC carcinogenesis, were chosen for further analyses (Supplementary Table S1). ASS1 protein levels in monolayer culture under the regular culture conditions and upon arginine deprivation were initially compared in the absence or presence of citrulline (0.05 mM or 0.4 mM). Then our findings were verified in a 3-D environment.

### CRC cells with CpG methylator phenotype upregulate ASS1 upon arginine deprivation

**Figure 2:**
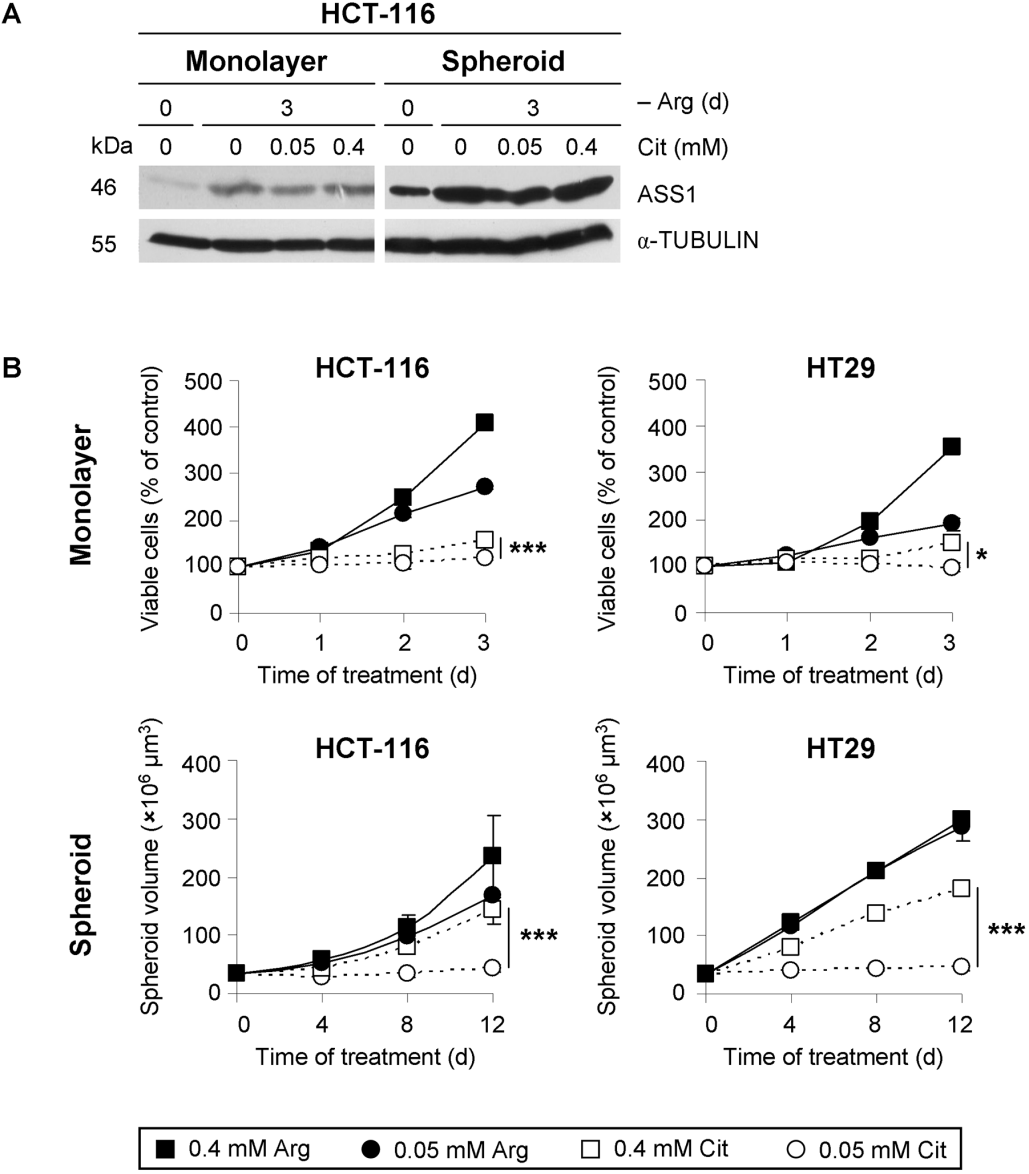
Physiological citrulline concentration supports CRC cell growth neither in monolayer nor in spheroid culture despite upregulation of ASS1 protein expression (**A**) Representative Western blots of protein extracts from monolayer and spheroid cultures of HCT-116 cells harvested before (day 0) or after a 3-day exposure to arginine-free (−Arg) medium alone or supplemented with the indicated concentrations of citrulline (Cit) are shown. Total protein extracts were probed with an anti-ASS1 antibody. α-TUBULIN was used as loading control. (**B**) HCT-116 or HT29 cells in monolayer or spheroid culture were incubated for the indicated periods of time in –Arg medium supplemented with Arg or Cit to final concentrations of 0.05 or 0.4 mM. Monolayer cell growth was assessed by the quantification of viable cells via an MTT assay in three independent experiments. Spheroid growth was determined by calculation of spheroid volume from the measured diameters of spheroids. Each data point presents the average volume (± SE) of at least 30 spheroids obtained in one experiment. This result was verified by an independent experimental series.

In agreement with our previous data [26], monolayer HCT-116 cells with low basal ASS1 protein level upregulated its expression when exposed to arginine-free conditions alone (Figure 2A). In spheroid culture, HCT-116 cells were characterized by a substantially higher basal ASS1 expression level than in monolayer culture. However, exposure of spheroids to arginine-free medium resulted in even higher ASS1 levels similar to the response in monolayer assay. Addition of citrulline to arginine-free medium at either concentration (0.05 or 0.4 mM) modulated ASS1 protein level neither in monolayer nor in spheroid culture. Virtually identical observations were documented for another CRC cell line HT29 (Supplementary Figure S1).

Since multicellular organization of spheroids might affect the pattern of ASS1 distribution, we also allocated this protein in median sections of HCT-116 spheroids. Under regular culture conditions, ASS1 protein was mainly detected in the spheroid periphery where proliferating, well-oxygenated cells are located. Arginine deprivation resulted in an overall increase in ASS1 protein level, i.e. intracellular fluorescence signals were in general enhanced and ASS1 protein was detected in cells throughout the entire spheroid (Supplementary Figure S2). Taken together, our observations verify that CRC cells with a CpG methylator phenotype are still capable to adapt expression of *ASS1* gene and might thus utilize citrulline as a precursor in the case of therapeutically-driven arginine deficiency.

### Citrulline concentration defines the growth rate of CRC cells in the absence of arginine

Upregulation of ASS1 in the absence of arginine implies that CRC cells can efficiently convert citrulline to compensate for a growth inhibitory effect of arginine deprivation-based therapy. However, even a hyperphysio-logical citrulline concentration (0.4 mM) only partially supported HCT-116 and HT29 cell growth both in monolayer and spheroid cultures (Figure 2B). The apparently stronger compensatory effect of 0.4 mM citrulline on spheroid volume growth as compared to the growth rate in monolayer culture correlated with the higher level of ASS1 protein detected in 3-D *vs*. 2-D environment under these conditions (Figure 2A). By contrast, the physiological citrulline level (0.05 mM) did not at all support cell proliferation in the absence of arginine, which was true for both monolayer and spheroid cultures of HCT-116 and HT29 cells. Our data suggest that high amounts of citrulline are crucial to support proliferation of arginine-starved CRC cells in monolayer and spheroid cultures; despite the enhanced ASS1 expression, physiological citrulline concentration is not sufficient for CRC cell growth in arginine-free environment.

### Substitution of arginine by citrulline decelerates cell cycle progression

**Figure 3:**
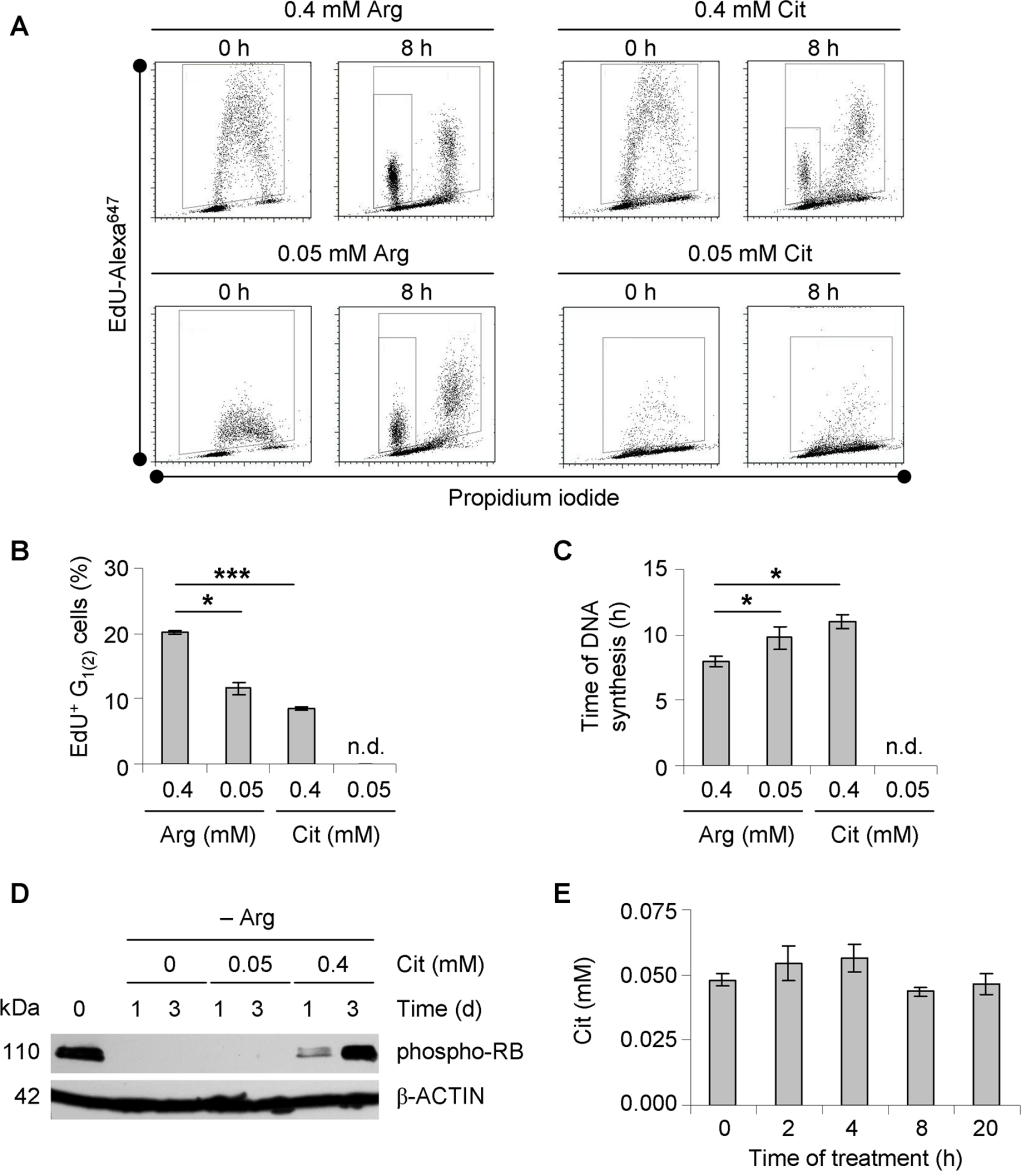
Substitution of arginine to citrulline affects cell cycle progression of CRC cells (**A**) Representative dot blot diagrams from EdU pulse-chase experiments are shown. Monolayer HT29 cells were pre-incubated in –Arg medium supplemented with Arg or Cit at 0.05 or 0.4 mM before EdU pulse-labeling to allow cell cycle adaptation to the dietary conditions. Total time of treatment in specific media was 72 h. Data obtained after a chase period of 0 and 8 h are documented. (**B**) Percentages of EdU-positive HT29 cells treated as described in (A), which had entered the subsequent second G_1_-phase (G_1(2)_) during the 8 h chase period are shown (mean ± SE of two independent experiments, n.d. – not detected). (**C**) Time of DNA synthesis as calculated from EdU pulse-chase experiments according to (A) is documented. n.d. – not detected. (**D**) Representative Western blots of protein extracts from monolayer HT29 cells maintained in –Arg medium alone or supplemented 0.05 or 0.4 mM Cit for 1 or 3 days are shown. Total protein extracts were probed with an anti-phospho-RB antibody. β-ACTIN was used as loading control. (**E**) Cit concentrations in the supernatant of monolayer HT29 cells incubated in –Arg medium with 0.05 mM Cit were measured by LC-MS at indicated time points and documented.

To better understand the regulation of growth retardation when extracellular arginine is substituted for citrulline (Figure 2B), dynamic cell cycle analysis by pulse-labeling with nucleotide analog EdU was performed. As shown in Figure 3A, EdU signals in monolayer HT29 cells exposed to 0.4 mM arginine or citrulline were analogous directly after the pulse. However, EdU uptake clearly dropped in cells maintained under the lower concentrations of either amino acid. The difference in EdU incorporation reflected the rate of DNA synthesis in cells pre-exposed to the specific media. Quantification of the EdU-positive cell fraction, which proceeded to a subsequent G_1_-phase (G_1(2)_) over a period of 8 h after labeling, allowed the tracing of cell cycle progression under different experimental settings. With this approach, we could clearly show that cell cycling is critically slowed in the presence of 0.05 mM as opposed to 0.4 mM arginine (Figure 3B). However, even cells maintained under low arginine conditions were proliferating and the G_1(2)_ cell fraction was still much higher than in the setting where arginine was substituted for hyperphysiological citrulline (0.4 mM) (Figure 3B). Reduction of the citrulline level to 0.05 mM completely abrogated cell cycling as could be concluded from the lack of a G_1(2)_-population. S-phase durations calculated from the experimental series for the other conditions underline this observation (Figure 3C). Consistent with this, Western blot data showed that 0.05 mM citrulline was not enough for arginine-starved HT29 cells to restore RB phosphorylation which is necessary for G_1_-S transition (Figure 3D).

The inability of 0.05 mM citrulline to support cell growth under arginine deprivation *in vitro* may be due to its rapid uptake and utilization, leading to an expeditious lack of citrulline in the culture medium. This would not reflect the *in vivo* situation where the concentration of citrulline is similarly low, but quite stable [27]. By determining the citrulline concentration in the medium over time, we could show that its level remains unchanged after the shift to arginine-free medium plus 0.05 mM citrulline over a period of at least 20 h (Figure 3E). This represents the time interval when the amount of proliferating HT29 cells decreased most rapidly (Supplementary Figure S3A). In fact, even daily medium refreshment (arginine-free with 0.05 mM citrulline) did not perpetuate proliferative activity of HT29 cells (Supplementary Figure S3B). Altogether, these results verify that arginine deprivation causes a cell cycle arrest, which cannot be overcome by physiological citrulline concentration in the tested CRC cells.

### Citrulline in arginine-devoid conditions cannot impede canavanine-induced apoptosis

We previously reported that the natural arginine analog canavanine potently enhances arginine deprivation-induced apoptosis in human cancer cells [23]. The impact of citrulline in this scenario is not yet known.

**Figure 4:**
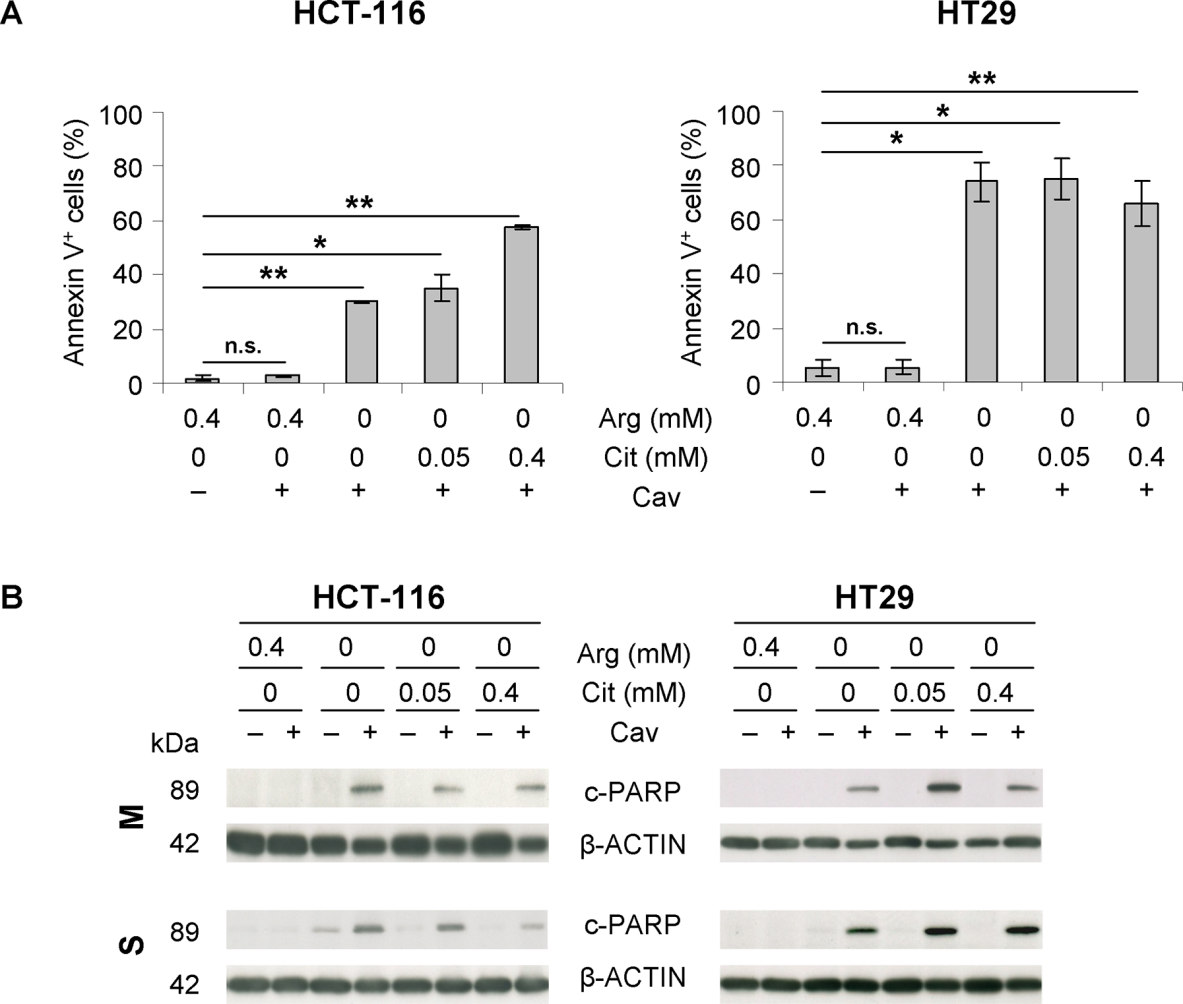
The combination of arginine deprivation with canavanine treatment triggers apoptosis in CRC cells regardless of citrulline supplementation (**A**) Fractions of apoptotic, annexin V-positive cells after 3 days of treatment with 0.1 mM canavanine (Cav) in –Arg medium with or without Cit compared to the respective treatment in arginine-rich medium (0.4 mM Arg) are shown. Cells maintained in arginine-rich medium without Cav served as control. (**B**) Representative Western blots of protein extracts from monolayer (M) and spheroid (S) cultures of HCT-116 and HT29 cells treated as described in (A) are shown. Protein extracts were probed with an anti-cleaved PARP (c-PARP) antibody; ß-ACTIN was used as loading control.

As shown in Figure 4A, both cell lines demonstrated massive apoptotic cell death in monolayer culture when exposed to 0.1 mM canavanine in the absence of arginine; this effect was independent of the presence of extracellular citrulline. HCT-116 cells turned out to be more resistant to this combination treatment than HT29 cells, as evident from the annexin V-positive cell fractions (~30–50% *vs*. ~70%; Figure 4A). PARP protein cleavage confirmed this finding and further revealed that only arginine, but not its precursor citrulline, is capable of preventing canavanine-induced apoptosis as verified in protein extracts from both monolayer and spheroid cultures (Figure 4B). These data imply that the combination treatment induces apoptosis in CRC cells both in a 2-D and 3-D environment regardless of citrulline availability.

### Citrulline can improve regrowth of CRC spheroids after the combined treatment

**Figure 5:**
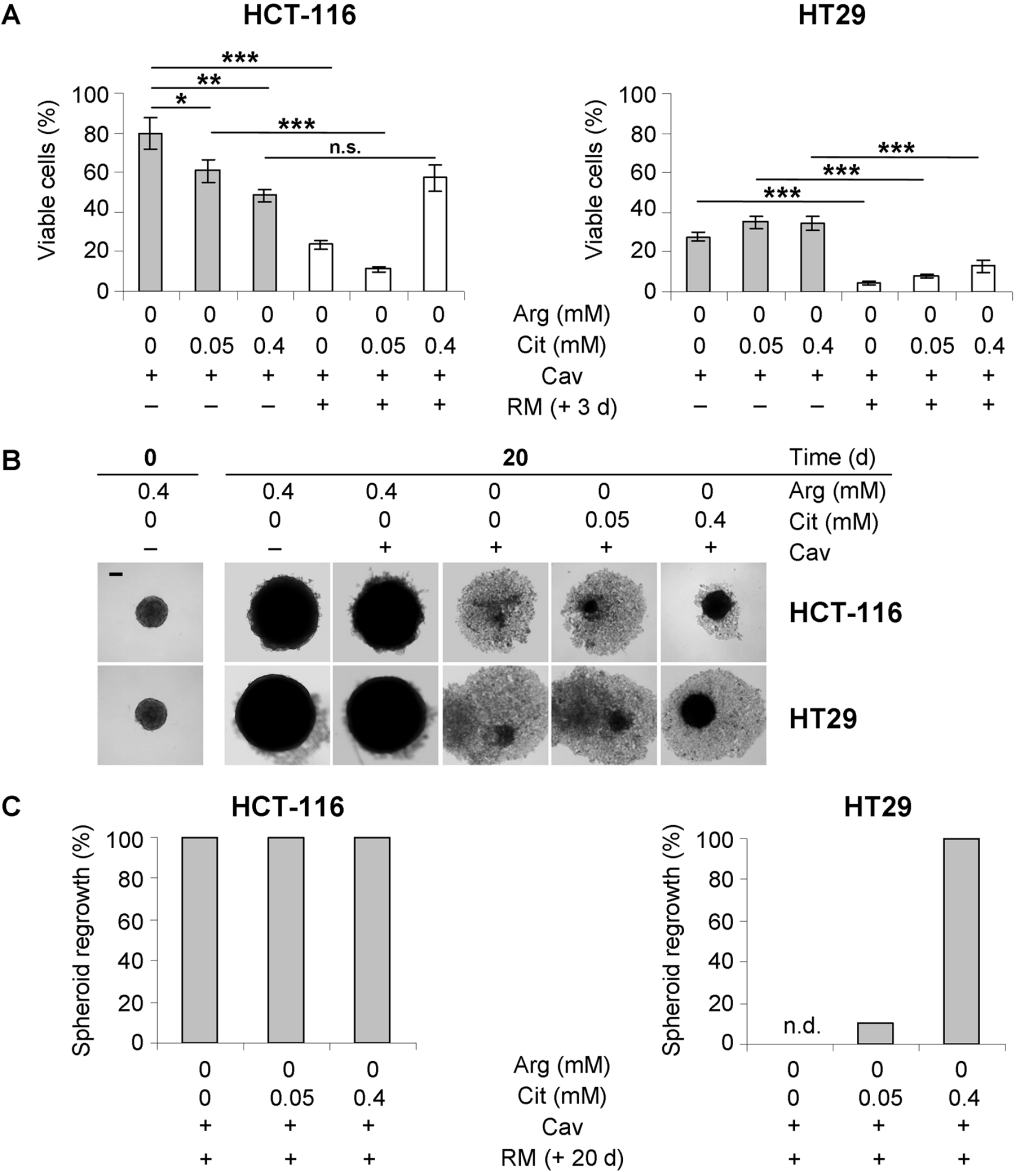
Only hyperphysiological citrulline can support regrowth of CRC spheroids after combined treatment with canavanine under arginine-deprived conditions (**A**) Monolayer HCT-116 and HT29 cells were exposed to –Arg medium containing 0.1 mM Cav with or without Cit supplementation for 3 days (grey bars). Afterwards, these specific media were exchanged to regular medium (RM), and the cells were allowed to grow for additional 3 days (white bars). Each data point presents the average number of viable cells (± SE) of three independent experiments. (**B**) Representative phase contrast images of HCT-116 and HT29 spheroids before treatment (day 0) and after 20 days of treatment with Cav in the specific media are shown. Scale bar = 200 μm. (**C**) The fractions of HCT-116 and HT29 spheroids that restored growth after 20 days of exposure to the combination treatment as described in (A) followed by an additional 20 days in RM post-treatment are shown. Each column represents data from at least 30 spheroids obtained in one experiment. n.d. – not detected.

In the absence of arginine, canavanine not only induces programmed cell death in cancer cell subpopulations, but also severely interferes with growth recovery of the remaining membrane-intact cells after the termination of treatment [18, 23]. We therefore evaluated the impact of citrulline supplementation on the regrowth potential of arginine-starved, canavanine-treated CRC cells. MTT viability data in monolayer cultures confirmed the observation that HCT-116 cells are more resistant to the combined treatment than HT29 cells (Figure 5A). However, citrulline at concentrations of 0.05 or 0.4 mM failed to increase the viable cell fraction upon combination treatment in both CRC cell line models. Moreover, the cells did not recover monolayer cell growth, but rather continued to die independently of citrulline availability, as evident from the ongoing decrease in the number of viable cells detected 3 days after cessation of treatment (Figure 5A).

In 3-D spheroid culture, 0.1 mM canavanine did not alter growth dynamics and integrity of CRC spheroids in an arginine-rich environment (Figure 5B). In the absence of arginine, however, canavanine not only completely abrogated spheroid growth, but also caused a severe spheroid disintegration, which is consistent with our previous findings [18]. Substitution of arginine by 0.4 mM citrulline during canavanine treatment did not support spheroid growth, but partially preserved spheroid integrity. By contrast, physiological citrulline level was not sufficient to prevent the canavanine-induced disruption of spheroids (Figure 5B). Staining with propidium iodide revealed that the majority of the cells (≥ 80%), which had detached from the spheroids, was membrane-defect in all experimental settings (data not shown).

Spheroid monitoring post-treatment clarified that the regrowth potential of HT29 spheroids was entirely abrogated after the exposure to 0.1 mM canavanine for 20 days under arginine-free conditions (Figure 5C). At the same time, supplementation of arginine-devoid medium with 0.05 mM citrulline resulted in a spheroid growth recovery of about 10%, while supplementation with 0.4 mM citrulline fully restored the regrowth capacity of treated HT29 spheroids. HCT-116 spheroids were exceptionally resistant to the combination of arginine deprivation and canavanine, as all of them restored growth even after 20 days of treatment (Figure 5C). The fact that CRC cells grown as spheroids showed lower sensitivity to the combination of arginine starvation and canavanine exposure than the respective monolayer cultures could be explained by differences in mTOR-mediated signaling in the 3-D *vs.* 2-D environment upon such treatment. In both HCT-116 and HT29 models, exposure to 0.1 mM canavanine in arginine-free medium led to an inhibition in the activity of the mTOR complex 1 (mTORC1) in spheroid but not monolayer cultures as manifested by the dephosphorylation of mTORC1 downstream targets, i.e. ribosomal protein S6 and eukaryotic translation initiation factor 4E binding protein 1 (4E-BP1) (Supplementary Figure S4). Reduced efficacy might thus be attributed to translation inhibition through mTOR signaling in in 3-D culture resulting in a diminished canavanine incorporation into nascent proteins, which is considered as the main route of its cytotoxicity [28].

Taken together, our data suggest that CRC cells are more efficiently protected by (hyperphysiological) citrulline from arginine deprivation-related anticancer activities of canavanine in the 3-D environment than expected from classical 2-D monolayer assays.

### Citrulline does not prevent radiosensitization by arginine deprivation and canavanine

**Figure 6:**
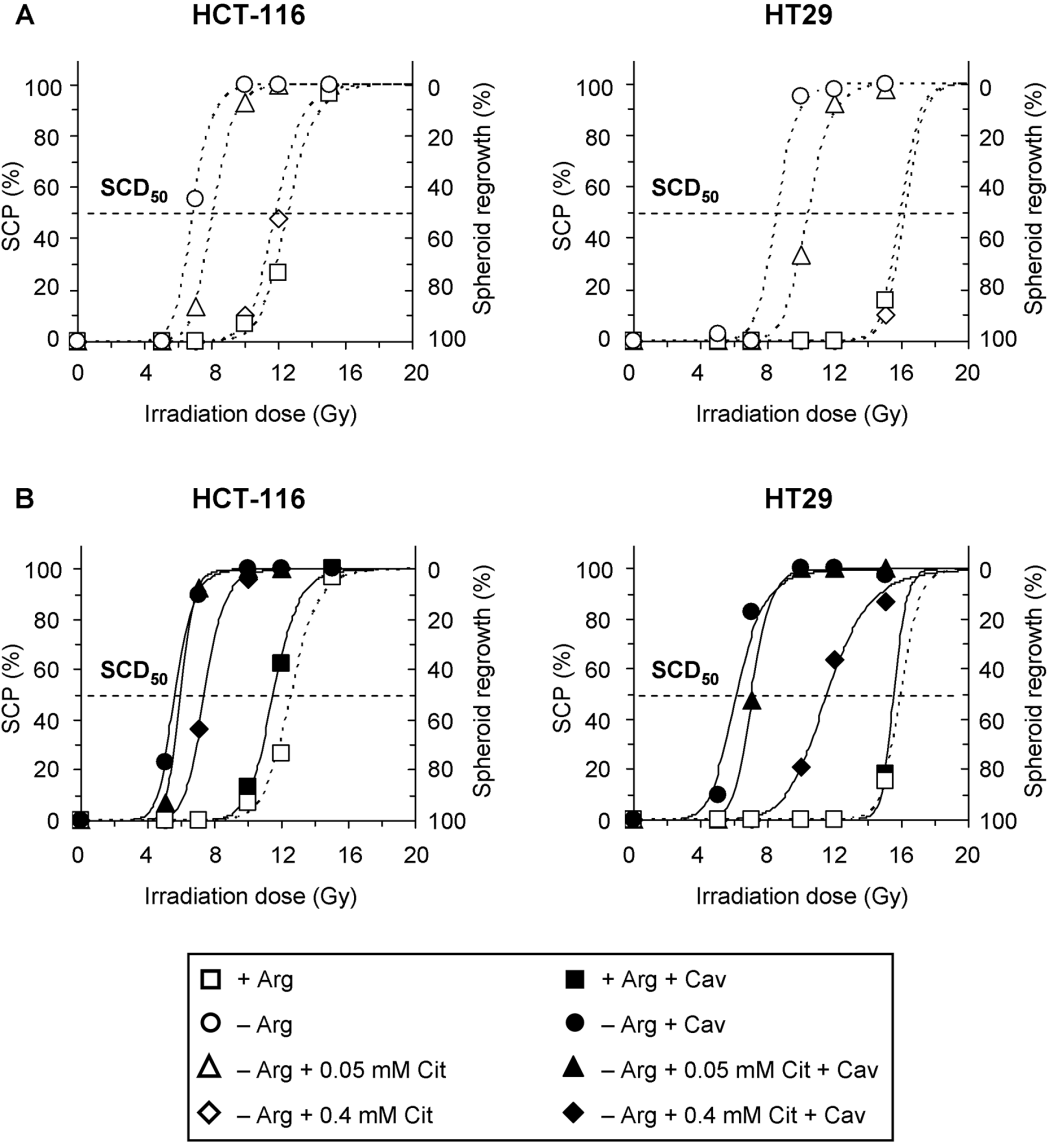
Combination of arginine deprivation with canavanine sensitizes CRC spheroids to irradiation regardless of citrulline supplementation Four days after inoculation, HCT-116 and HT29 spheroids with a mean diameter of ~400 μm were exposed to specific treatment conditions without (**A**) or with (**B**) canavanine for 5 days prior to single dose irradiation. The percentage of spheroids that lost regrowth capacity (spheroid control probability, SCP) is shown as a function of irradiation dose. *n* = 30–40 individual spheroids per condition were monitored over a period of 60 days post-treatment. Treatment conditions: –Arg – arginine-free medium; +Arg medium was obtained by supplementation of –Arg conditions with 0.4 mM Arg and used as control; Cav – 0.1 mM Cav; SCD_50_ – spheroid control dose_50_.

Finally, we addressed the impact of citrulline on the radiosensitizing potential of arginine deprivation therapy alone and when combined with canavanine. Dose response curves showing the regrowth probabilities of HCT-116 and HT29 spheroids as a function of irradiation dose are documented in Figure 6. From the curve fittings, the SCD_50_ as the dose leading to a loss of regrowth capacity in 50% of the spheroids was calculated for each treatment regime (Table 1, Supplementary Table S2). HCT-116 spheroids were generally more sensitive to irradiation than HT29 spheroids (SCD_50_: 12.8 Gy *vs*. 16.0 Gy, *p <* 0.001). Pre-exposure of spheroids to arginine deprivation reduced SCD_50_ values to 7.0 Gy for HCT-116 and 9.5 Gy for HT29 spheroids, which resulted in a dose reduction factor of 1.8 and 1.9 when compared to the respective arginine-containing control conditions, reflecting a massive radiosensitization. Citrulline at 0.4 mM in arginine-free medium escalated SCD_50_ back to values compatible with those observed in the presence of arginine for both CRC spheroid models. Thus, arginine deprivation-induced radiosensitization was virtually abolished by hyperphysiological citrulline concentration, whereas physiological citrulline level (0.05 mM) only marginally counteracted the radiosensitizing effect of arginine deprivation (Figure 6A, Table 1).

**Table 1:** SCD_50_ values for HCT-116 and HT29 spheroids, which had been exposed to different treatment conditions for 5 days before irradiation

Treatment	HCT-116		HT29	
	SCD_50_, Gy	DRF [95% CI]	SCD_50_, Gy	DRF [95% CI]
+ Arg (control)	12.8	–	16.0	–
– Arg	7.0	1.82 [1.66–2.00]	9.5	1.86 [1.75–1.97]
– Arg + 0.05 mM Cit	8.2	1.54 [1.43–1.66]	10.4	1.53 [1.47–1.60]
– Arg + 0.4 mM Cit	12.1	1.06 [1.00–1.13]	16.4	n.s.
+ Arg + Cav	11.6	1.10 [1.03–1.16]	15.6	n.s.
– Arg + Cav	5.7	2.24 [2.06–2.42]	6.2	2.61 [2.45–2.79]
– Arg + 0.05 mM Cit + Cav	6.0	2.11 [1.97–2.26]	7.0	2.27 [2.11–2.42]
– Arg + 0.4 mM Cit + Cav	7.3	1.69 [1.57–1.81]	11.3	1.38 [1.31–1.46]

+Arg – control medium, arginine-free medium supplemented with 0.4 mM arginine; –Arg – arginine-free medium; Cit – citrulline; Cav – 0.1 mM canavanine; SCD_50_ – spheroid control dose 50; DRF – dose reduction factor with 95% confidence interval (CI) relative to SCD_50_ in control medium; n.s. – not significant.

Application of 0.1 mM canavanine alone - in contrast to arginine deprivation alone - did not sensitize spheroids to irradiation (Figure 6B, Table 1, Supplementary Table S2). The combination of canavanine with arginine deficiency, however, had a synergistic impact on radioresponse, resulting in SCD_50_ values as low as 5.7 Gy for HCT-116 and 6.2 Gy for HT29 spheroids. Thus, radiosensitivity was increased by a factor of 2.2 and 2.6, respectively, as compared to spheroids exposed to canavanine in the presence of arginine. The SCD_50_ data documented in Table 1 further illustrate that even hyperphysiological citrulline only partially protected CRC spheroids from radiosensitization caused by canavanine in an arginine-free environment, while physiological citrulline level did not generate any radioprotecting potential in this context.

Our data clearly demonstrate that citrulline at physiological concentration cannot withstand the radiosensitizing potential of arginine deprivation combined with canavanine treatment. Notably, the treatment benefit of combining arginine starvation, canavanine and radiotherapy was particularly high in the HCT-116 spheroid model, which was initially less susceptible to canavanine upon the lack of arginine than the HT29 model.

### Canavanine treatment upon arginine deprivation is not toxic for normal colon epithelium

The major drawback of anticancer treatments is their toxicity for normal, non-malignant cells. Therefore, we next elucidated if the combination of arginine deprivation with canavanine treatment affects the viability of non-malignant colon epithelium. The 3-D culture of organoids/colonospheres was applied because it better resembles normal tissue milieu and supports proliferation of epithelial cells from intestine and colon [29]. Since the maintenance of these normal colon cell cultures requires a particular cell culture medium, colonospheres of HT29 cells were studied in parallel. In this experimental setup arginine deprivation was achieved via the addition of the arginine-degrading enzyme rhARG. Arginine-deprived medium was supplemented with physiological concentrations of citrulline (0.05 mM) alone or together with low dose canavanine (0.1 mM).

**Figure 7:**
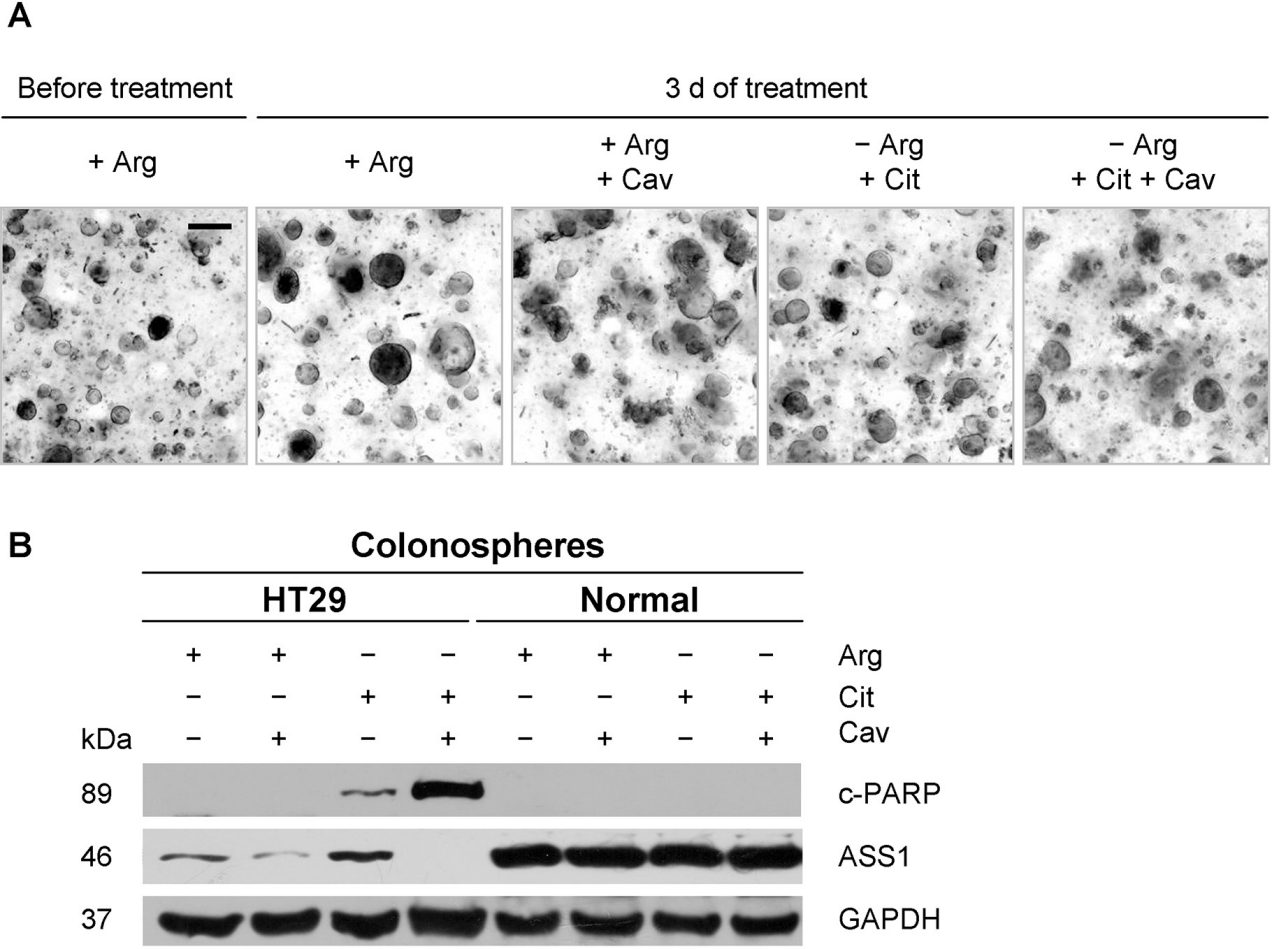
Combination of arginine deprivation with canavanine does not induce apoptosis in normal colon epithelial cells in 3-D organoid/colonosphere culture (**A**) Representative, deconvoluted z-stack phase contrast images of normal colonospheres before and after 3 days of exposure to the indicated treatment conditions; −Arg environment was achieved by supplementation of the regular for colonospheres cell culture medium with 2 U/ml rhARG. Cit and Cav were supplemented to final concentrations of 0.05 mM and 0.1 mM, respectively. Scale bar = 200 μm. (**B**) Western blots of protein samples extracted from HT29 and normal colonosphere cultures treated as described in (A); protein extracts were probed with anti-c-PARP and anti-ASS1 antibodies; GAPDH was used as loading control.

Z-stack phase contrast images document the expected increase in size of both normal (Figure 7A) and HT29 colonospheres (data not shown) over a period of 3 days under regular culture conditions. This was only marginally reduced under 0.1 mM canavanine treatment (Figure 7A). Upon arginine deprivation, 0.05 mM citrulline partly supported the growth of normal colonospheres (Figure 7A), but not HT29 colonospheres. Combination of the arginine-deprived environment with low dose canavanine treatment did not reduce normal colonosphere integrity (Figure 7A).

Western blot analyses show that ASS1 expression and its regulation clearly differ between normal and malignant colonospheres (Figure 7B). Basic ASS1 protein level was much higher in normal colon epithelial cells, while upregulation upon arginine withdrawal as well as massive downregulation in the presence of canavanine was only seen in HT29 cell population. In the presence of 0.05 mM citrulline, neither arginine deprivation nor its combination with 0.1 mM canavanine resulted in PARP fragmentation in normal colonospheres, while apoptosis was clearly induced in HT29 cultures under the identical conditions as visualized by c-PARP protein accumulation (Figure 7B). The data on apoptosis induction in HT29 colonospheres correlates well with the results obtained for HT29 spheroids maintained in arginine-free medium supplemented with canavanine (Figure 4B). We therefore conclude that enzymatic depletion of arginine by rhARG in combination with low dose canavanine exhibits selective anticancer activity and does not affect the viability of normal colon epithelial cells.

## DISCUSSION

It is widely anticipated that arginine deprivation-based therapy is exclusively applicable for ASS1-negative tumor entities as they cannot rely on plasma citrulline for arginine synthesis [4, 6]. However, even tumors, which are initially sensitive to arginine deficiency, might acquire resistance during therapy [30, 31]. Several studies utilizing one of two therapeutic arginine-degrading enzymes, namely ADI, ascribed such resistance to upregulation of *ASS1* gene expression in treated malignant cells [30, 32]. Indeed, the highly variable expression of the *ASS1* gene and in particular its upregulation under various stress conditions, such as arginine restriction [3, 30, 33] and chemotherapy [25], or during the course of carcinogenesis [24, 34, 35], is a major challenge for patient stratification and therapy optimization. In this context, attention should also be paid to the 10-fold increase in citrulline concentration, which is, alongside with ammonia, the major product of ADI-mediated arginine hydrolysis [8], and could be (re-)converted to arginine via the ASS1-ASL enzyme axis.

Until now, CRCs were excluded as therapy responders based on an early work of Dillon *et al*, who demonstrated ASS1 expression in 46 out of 47 human CRC tissue samples CRCs [7]. Interestingly, we found 9 out of 16 established human CRC cell lines to have low or undetectable levels of ASS1 protein under the regular culture conditions. Most of these cells have a CpG methylator phenotype, which is known to epigenetically silence *ASS1* gene expression [9, 24, 25]. Our data verify that CRC cell lines with low basal ASS1 protein levels can accumulate ASS1 enzyme under arginine-deprived conditions in monolayer culture as shown for the genetically distinct CRC models HCT-116 and HT29 (present study and [26]). These CRC cell lines represent both pathways of CRC carcinogenesis (microsatellite *vs*. chromosomal instability) [36, 37], differ in their genetic profile, e.g in *TP53* status (wild type *vs*. mutated) [37] which contributes to the regulation of cellular metabolism and radioresponse [38], and supposedly vary in their sensitivity to arginine deprivation in monolayer culture (resistant *vs*. sensitive cell line) [18]. Hence, they are considered excellent models to study the phenomenon of ASS1-related resistance in more detail and address the role of extracellular citrulline in the anticancer potential of arginine deprivation therapy alone or in combination with irradiation and other treatment strategies. The cells' ability to form 3-D multicellular tumor spheroids as an *in vitro* assay of intermediate complexity between standard monolayer culture and *in vivo* experiments [39, 40] turned out to be of particular interest as both cell lines showed enhanced ASS1 protein expression when grown in a 3-D environment. This could explain the discrepancy between the ASS1 expression profile in cell lines and in CRC biopsy specimen [7] underpinning that 3-D spheroids better reflect the *in vivo* situation than monolayer cultures. Anyways, the process of ASS1 upregulation upon arginine withdrawal was still functioning in the 3-D cellular context.

Our experimental series further substantiated that the capacity of cancer cells to utilize citrulline for growth induction in the absence of arginine depends not only on ASS1 protein level but also on citrulline availability. This is especially intriguing not only in the light of the therapeutic enzymes in clinical trials, i.e. ADI, but not rhARG, may increase citrulline blood level, but also with respect to recent findings on amino acid availability in primary CRC tissue and distant metastases [41, 42]. There, arginine and citrulline concentrations in blood turned out to be significantly lower in cancer patients than in healthy volunteers, while the levels of these amino acids in CRC tissues were much higher than in the matched adjacent normal colon tissues. The observation clearly indicates an enhanced uptake of arginine and citrulline by CRC cells and implies that this tumor entity might be more sensitive to arginine deprivation than expected from the ASS1 profile. Indeed, a similar decline in the amount of circulating arginine was observed in patients with liver tumors, well-known for their arginine auxotrophy [10, 41]. Of note, even the higher concentrations of citrulline recorded in those CRC studies [41, 42], i.e. ~0.01 mM, were below the physiological citrulline level in the human bloodstream [27, 43]. It is thus worth to emphasize that physiological citrulline (0.05 mM) was unable to support proliferation of CRC cells under arginine-deficient conditions both in monolayer as well as in spheroid culture despite any therapy-induced ASS1 upregulation.

In the present study, it was shown that CRC cell growth can partly be rescued from arginine starvation by a hyperphysiological citrulline concentration. Citrulline may thus also diminish the cytotoxicity of the arginine analog canavanine under arginine-deficient conditions. The mechanism responsible for canavanine-induced cell death was determined by us and others as mitochondrial caspase-dependent apoptosis [3, 20]. However, induction of apoptosis correlated with growth restoration potential only in monolayer, but not in spheroid culture. Moreover, the rate of apoptosis in cell cultures exposed to canavanine in the absence of arginine appeared to be entirely unrelated to the amount of extracellular citrulline in both 2-D and 3-D assays. Spheroid cultures of CRC cells were in general more resistant to the proposed combination treatment than monolayer cultures, and only benefited from an excess of citrulline as shown by some increase in the spheroid regrowth probability.

Our data indicate that spheroid recurrence is not exclusively defined by the portion of dead cells after exposure to the proposed metabolic treatment, but rather by internal properties of the membrane-intact, surviving (non-apoptotic) cell population. One of such properties of the surviving cells could be translation regulation. In this context, we have recently demonstrated that the mTOR pathway contributes to the differential sensitivity of HCT-116 and HT29 cells to the lack of arginine [44], i.e. the more resistant HCT-116 cells rapidly and persistently inactivated mTORC1 upon arginine deprivation while the sensitive HT29 cells exhibited only a transient (up to 8 h) inhibition of mTORC1. Findings in the present study reveal that the enhanced resistance to canavanine treatment in an arginine-deficient environment of spheroid as compared to monolayer CRC cells might also be explained by a strong downregulation of translation-related mTOR signaling. Nonetheless, the potential of canavanine to interfere with other arginine-dependent processes needs to be carefully addressed in prospective studies.

The major pathway of canavanine metabolism in mammals is considered to be the urea cycle, where it is hydrolyzed by arginase to canaline and urea [45]. The treatment with canavanine is thus expected to severely affect liver as the main organ functionally requiring the urea cycle. However, first *in vivo* experiments imply that canavanine toxicity and damage are not restricted to hepatocytes but rather seen in the pancreas, where the drug predominantly accumulated [46]. The mechanism of this accumulation is still unclear and needs to be elucidated before translation into the clinics. However, it is likely to assume that the liver urea cycle would not be severely and permanently perturbed by low-dose canavanine administration. Indeed, we have shown that canavanine manifests its anticancer potential at significantly lower doses when combined with arginine deprivation [23] and therefore expect a reduced general toxicity for the organism.

To verify this speculation, we examined normal human colon epithelial cells maintained in a sophisticated 3-D organoid/colonosphere culture system [29]. The situation *in vivo* was simulated by the application of rhARG [47] to reduce extracellular arginine concentration and supplementation of the cell culture medium with the physiological amount of citrulline. Under such conditions and with the low dose of interest for combination with radiotherapy, we observed no canavanine toxicity in normal colon epithelial cells, as concluded from preserved colonosphere integrity and absence of c-PARP signal. The combination of arginine starvation with canavanine treatment is currently evaluated *in vivo* both by our group and others (https://ash.confex.com/ash/2015/webprogram/Paper83756.html) [48] and will bring further insight into the anticancer efficacy of the proposed therapy.

The sensitivity of cancer cells to irradiation depends on various intrinsic and extrinsic factors. Amongst others, starvation for arginine reduces the level of its derivatives (polyamines and nitric oxide), which have been known to modulate radioresponse for a long time [49, 50]. Other mechanisms via ER stress response have come into focus more recently [26]. A limited number of studies reported that ADI-induced arginine depletion enhanced radiosensitivity, i.e. in MCF-7 human breast carcinoma cells *in vitro* [51] and in neuroblastoma tumor xenografts in mice [52]. The impact of *ASS1* expression and/or citrulline availability on *in vivo* cell sensitivity to irradiation was not evaluated. However, Park and colleagues [52] reported that radiosensitization of MCF-7 cells in monolayer culture was due to an aberrant expression of cell cycle proteins and independent of very high citrulline levels (1 mM) produced by ADI. Our own data reported herein clearly demonstrate that only physiological levels of citrulline do not interfere with arginine deprivation-driven radiosensitization in CRC cells when grown under 3-D conditions, while a hyperphysiological citrulline concentration even lower than 1 mM (0.4 mM) can abrogate the radiosensitizing effect. The discrepancy might causally relate to the decreased susceptibility of spheroid cultures to various stress factors as reported here and in [40, 53] and the enhanced ASS1 level in 3-D *vs*. 2-D culture. Importantly, co-application of arginine deprivation with low-dose canavanine treatment was super-additive with respect to radiosensitization and also diminished the radioprotective effect of elevated citrulline in both CRC models. Our data thus suggest that the combination of arginine starvation with canavanine treatment and irradiation may provide a strategy of clinical potential for treating tumor types with inducible ASS1 expression. Application of rhARG instead of bacterial ADI might be considered for this treatment approach, as the former should not increase the concentration of citrulline in plasma and has lower affinity to canavanine [54–56].

In conclusion, we have demonstrated here for the first time that: 1) physiological levels of the arginine precursor citrulline are not sufficient to recover CRC cell growth upon arginine deprivation regardless of the basal level of ASS1 protein expression in the cancer cells and its inducibility; 2) not even an excess of citrulline, not to mention its physiological concentration, can abrogate the radiosensitizing effect of arginine deprivation combined with canavanine treatment.

## MATERIALS AND METHODS

### Cell lines and routine culturing

Human CRC cell lines were obtained from the ATCC (Manassas, VA, USA). Cell lines were mycoplasm-free as regularly tested with the Mycoplasma Detection Kit MycoAlert (Cambrex Bio Science, Nottingham, Ltd, UK) and a mycoplasma-specific PCR (Applichem, Darmstadt, Germany). Cell line authentication was performed with multiplex PCR kits, i.e. Mentype^®^ Nonaplex^QS^ Twin (Biotype AG) and the PowerPlex^®^ 16 System (Promega GmbH, Mannheim, Germany) at the Institute of Legal Medicine (TU Dresden, Germany) as reported previously [18]. Cells were routinely maintained in DMEM containing 1 g/l glucose, 3.7 g/l NaHCO_3_ and 25 mM HEPES, which was supplemented with 100 U/ml penicillin, 100 μg/ml streptomycin and 10% FBS prior to use (herein referred to as “regular medium”). Cells were kept at 37^°^C in a humidified atmosphere containing 8% CO_2_. All reagents for cell culturing were purchased from PAN-Biotech (Aidenbach, Germany). Single-cell suspensions for passaging and experimental setup were obtained from exponentially growing monolayer stock cultures by mechanic and enzymatic means using 0.05% trypsin/0.02% EDTA in PBS.

### Arginine deprivation-based treatment

Arginine deprivation was achieved by using DMEM-based arginine-free (−Arg) medium. The arginine precursor citrulline (Cit) was added to –Arg medium at concentrations of 0.05 mM (according to its physiological concentration in human blood/plasma) [27] or 0.4 mM (equimolar to the arginine content in regular medium). As controls, –Arg medium was supplemented with arginine at 0.05 mM or 0.4 mM concentrations. The natural arginine analog L-canavanine (Cav) (PubChem CID: 439202) was added at a concentration of 0.1 mM. To guarantee defined amounts of arginine in all experimental settings, media were supplemented with 10% of dialyzed FBS (depleted of molecules < 10 kDa, i.e. amino acids).

### Monolayer assays

Defined numbers of cells were seeded as single cell suspensions into appropriate culture plates using regular medium and allowed to attach overnight. Afterwards, cells were washed twice with PBS and exposed to specific dietary media (see above). Treatment conditions and incubation times differed for various experimental series and are documented in the results section. Viable cells were quantified upon dissociation via a CASY^®^ TTC cell analysis device (Roche Innovatis AG, Reutlingen, Germany) or compared with an MTT assay (Sigma-Aldrich) as detailed in [23]. Results are presented as percentage of viable cells relatively to untreated controls before medium exchange.

In monolayer growth restoration experiments, viable cells were assessed in one half of the samples directly after completion of the treatment using the MTT assay. The other half was re-supplemented with arginine to a final concentration of 0.4 mM (as in regular medium) and cell viability was again determined 3 days post-treatment.

### Spheroid growth assay

Spheroids were cultured in liquid overlay as described previously [57]. In brief, 1,000 HCT-116 or 1,500 HT29 cells were seeded into 1.5% agarose-coated 96-well plates in 200 μl of regular medium per well. At day 4 after inoculation, spheroids had reached a standard diameter of ~400 μm and were thoroughly washed in PBS to be transferred into the specific dietary media onto fresh agarose-coated plates. The agarose solutions for coating were prepared using the respective dietary or non-dietary DMEM-based media without serum. After defined periods of treatment, spheroids were collected and further processed, or the medium was supplemented with arginine to a final concentration of 0.4 mM. Spheroid growth was then monitored over a period of 20 days. A minimum of 30 spheroids was analyzed per experimental condition.

Spheroids were routinely fed twice per week by 50% medium renewal. Spheroid morphology and growth were recorded regularly using an Axiovert200M microscope equipped with an AxioCam MRm camera (Carl Zeiss MicroImaging, Heidelberg, Germany) as highlighted earlier [57].

### Spheroid radioresponse assay

HCT-116 and HT29 spheroids were transferred into the specific media (see above) and left for 5 days before single dose irradiation at 0–20 Gy (200 kV X-rays; 0.5-mm Cu filter; YxlonY.TU 320; Yxlon International, Germany). Afterwards, all media were supplemented with arginine to a final concentration of 0.4 mM and monitored regularly for another 60 days. Spheroid regrowth and spheroid control probability (SCP), respectively, was assessed as a function of the executed single irradiation dose for each type of treatment. The spheroid control dose 50 (SCD_50_), as the dose leading to a 50% loss of spheroid regrowth capacity, was calculated from the SCP curves fitted by logistic regression using the software STATA 11 (StataCorp LP, TX, USA).

### 3-D organoid/colonosphere culture from normal human colon epithelial crypts

Surgically resected colon tissue was obtained from patients at the University hospital Dresden who provided informed consent before surgery as approved by the local ethical committee. Normal colonic crypts were isolated and cultured as described previously [29]. In brief, crypts were embedded in Matrigel on ice (growth factor reduced, phenol red free; BD Biosciences) and seeded in 48-well plates (500 crypts per 20 μl of Matrigel per well). To ensure that CRC cell line data and effects in normal organoids/colonospheres do not artificially differ due to peculiarities of culture conditions, we also analyzed colonosphere cultures from HT29 cells (2.5 × 10^5^ cells per 20 μl of Matrigel per well). The Matrigel was polymerized for 10 min at 37^°^C, and then overlaid with 250 μl/well of human intestinal stem cell medium (HISC). HISC is an advanced DMEM/F12 medium supplemented with penicillin/streptomycin, 10 mM HEPES, 2 mM GlutaMAX, 1 × N2, and 1 × B27 (all from Invitrogen), with the following niche factors: 50 ng/ml mouse recombinant EGF, 100 ng/ml mouse recombinant noggin (Peprotech, Rocky Hill, NJ, USA), 10% R-spondin-1 conditioned medium, 50% Wnt-3A conditioned medium, 500 nM A83-01 (Tocris, Bristol, UK) and 10 μM SB202190 (Sigma-Aldrich). One day after inoculation, colonospheres were split in a 1:1 and 1:4 ratio for normal epithelial cells and HT29 cells, respectively. Upon two days of recovery, colonosphere cultures were exposed to either arginine-supplemented or arginine-deprived conditions for a 3-day period. Arginine deprivation in HISC medium was achieved by a 4–6 h pre-incubation with 2 U/ml rhARG at 37^°^C [47] while 0.05 mM Cit and/or 0.1 mM Cav were supplemented immediately before feeding the cultures for treatment.

Colonosphere cultures were imaged before and after treatment by taking z-stacks (30–40 images with a feed rate of 40 μm) using an AxioObserver (Carl Zeiss MicroImaging). After image acquisition, a deconvolution of the z-stacks was performed to reduce out of focus noise and obtain sharp 2-D images of high quality reflecting the entire depth of the matrix. For protein analyses in colonospheres before and after treatment, Matrigel was depolymerized by using the Matrigel Recovery Solution (Corning Inc., New York, NY, USA) as described by the manufacturer. the released colonospheres were washed and pelleted, and protein extraction plus Western blotting were performed as detailed below.

### Western blotting

Western blot analyses were conducted using whole cell protein extracts from 2-D and 3-D cultures according to an established protocol [18]. Primary antibodies detections were against ASS1 (Acris Antibodies, Herford, Germany), c-PARP, phospho-RB (both Cell Signaling Technology, Danvers, MA, USA), GAPDH (Santa Cruz Biotechnology, Heidelberg, Germany), β-ACTIN (Abcam, Cambridge, UK), and α-TUBULIN (Merck, Darmstadt, Germany). For detection, HRP-conjugated secondary antibodies (Dako, Hamburg, Germany) and a Western Blotting Luminol Reagent kit (Santa Cruz Biotechnology) were applied (see also Supplementary Table S3).

### Immunofluorescence in frozen spheroid sections

HCT-116 spheroids with diameter of ~400 μm were either left untreated (day 0) or exposed to arginine-free medium for 3 days. After collection at the indicated time points, spheroids were washed in PBS, snap-frozen in liquid nitrogen and embedded in tissue freezing medium (Leica Microsystems, Wetzlar, Germany). Spheroids cryosections (10 μm in thickness) were transferred onto slides and fixed in cold acetone for 10 min. Immunofluorescent staining was performed using a coverplate system (Thermo Fisher Scientific GmbH,). Unspecific binding sites were blocked with 0.5% goat serum in PBS. ASS1 was visualized in median spheroid sections using a monoclonal mouse anti-ASS1 antibody (Abcam) followed by Alexa^594-^conjugated goat anti-mouse secondary antibody (Invitrogen). Substitution of the primary antibody by an adequate isotype control antibody (Abcam) served as negative control (Supplementary Table S3). Nuclei were counterstained with 1 μM DAPI (Invitrogen) in PBS for 10 min at room temperature. Slides were mounted, stored overnight at 4°C, and imaged with an AxioImager M1 microscope (Carl Zeiss MicroImaging).

### Flow cytometry

For dynamic proliferation assessment, monolayer HT29 cells were pulse-chased with the thymidine analog 5-ethynyl-2′-deoxyuridine (EdU, Invitrogen) as previously described in [58]. In brief, 6 × 10^5^ exponentially growing cells per dish were exposed to specific dietary media for a total of 72 h. EdU pulse and chase periods were scheduled in a way that all samples could be seeded and harvested at the same time (Supplementary Figure S5). To ensure that cell cycling adjusted to the respective conditions, cells were incubated in specific media for at least 63 h before addition of 10 μM EdU for 1 h (pulse period). Afterwards, cells were washed with PBS and exposed to the fresh corresponding specific media for 0, 2, 4, 6, or 8 h (chase periods). EdU incorporation was detected following the protocol of the Click-iT EdU Flow Cytometry Assay Kit (Invitrogen). RNA was eliminated from the samples by incubation with RNase A (0.1 mg/ml, Invitrogen) at 37^°^C for 30 min. Propidium iodide (PI, Sigma-Aldrich) for stoichiometric DNA counterstaining was added at final concentration of 0.05 mg/ml directly before flow cytometric analysis.

For annexin V/PI staining, 1 × 10^6^ HCT-116 or HT29 cells were seeded per dish in regular medium. After 48 h, cells were washed with PBS and medium was changed to specific ones. The annexin V/PI apoptosis assay was performed after 72 h of incubation by the Annexin V-FITC Kit (Miltenyi Biotec, Bergisch Gladbach, Germany) according to the supplier's instructions.

Samples were analyzed using a BD FACS Canto II flow cytometer (Becton Dickinson, Heidelberg, Germany). 2 × 10^4^ or 3 × 10^4^ events were recorded for EdU incorporation and annexin V binding experiments, respectively. FlowJo version 7.6.4 (Tree Star, Ashland, TN, USA) software was used for all data analyses.

From EdU incorporation experiments, the DNA synthesis time (T_s_) was calculated as described by Begg *et al*. [59]. This method is based on the analysis of the progression of the EdU-positive cell population through the S-phase and calculation of a relative movement (RM) for the EdU-positive cell fraction according to:

$$\text{RM}=\frac{{\text{F}}_{\text{L}}-{\text{G}}_{\text{G}1}}{{\text{F}}_{\text{G}2}-{\text{F}}_{\text{G}1}}$$

F_L_ is the mean PI fluorescence of the EdU-positive cells that have not undergone mitosis, and F_G1_ and F_G2_ are the mean PI fluorescence signals of cells in G_1_/G_0_ and G_2_/M phases of the cell cycle, respectively. Finally, RM was plotted as a function of time, and T_s_ was calculated by linear regression as the time when RM equals 1.

### LC-MS for amino acid concentration measurement

Analysis of Cit concentrations in cell culture media was performed by Liquid Chromatography Mass Spectrometry (LC-MS; Agilent Technologies 1200 series and Triple Quad LC/MS 6410, both from Agilent, Boeblingen, Germany). Samples were harvested at indicated time points and stored at −80°C before analysis. After thawing, a defined amount of a deuterized internal Cit standard (L-citrulline-2,3,3,4,4,5,5-d7, CDN Isotopes, Essex, UK) was added to each sample. Deproteinization was performed using ice-cold acetonitrile/methanol 70/30, and samples were centrifuged for 5 min with 10,000 × g at 4°C. Supernatants were purged through a 0.2 μm filter and run on a HyperCarb column (Thermo Fisher Scientific GmbH) with a gradient of 97% of 0,1% TFA in H_2_O/3% acetonitrile to 100% acetonitrile. Single ion monitoring was performed in positive mode for m/z 176 for citrulline and m/z 183 for the deuterized isotope standard with a dwell time of 200 ms and a fragmentor voltage of 80 V. Product ion scans were recorded applying the conditions described above from 1 to 8 min with the precursor ions m/z 176 and m/z 183 using a collision energy of 25 eV (quantifier). Concentrations were determined by area under the curve calculations and correlation to standards.

### Statistical analyses

Data are presented as means ± SE from at least two independent experiments with at least three parallels for each experimental condition. *p* values were calculated by two-sided Student's *t* tests and values < 0.05 were considered significant. SCD_50_ values were tested for significant differences between treatment groups using a bootstrapping procedure. For each comparison, 2000 bootstrap samples of the dose-event data were created. For each sample, a logistic regression was performed per treatment group and the resulting difference in SCD_50_ was calculated using the STATA 11 software (StataCorp LP). Finally, the *p-value* was determined as two times the fraction of differences < 0 if most differences were > 0, or *vice versa*. The same bootstrapping procedure was applied to calculate the 95% confidence intervals of the SCD_50_ values.

## SUPPLEMENTARY MATERIALS TABLES FIGURES



## References

[1] Morris SM (2004). Enzymes of arginine metabolism. J Nutr.

[2] Curis E, Nicolis I, Moinard C, Osowska S, Zerrouk N, Benazeth S, Cynober L (2005). Almost all about citrulline in mammals. Amino Acids.

[3] Husson A, Brasse-Lagnel C, Fairand A, Renouf S, Lavoinne A (2003). Argininosuccinate synthetase from the urea cycle to the citrulline-NO cycle. Eur J Biochem.

[4] Qiu F, Huang J, Sui M (2015). Targeting arginine metabolism pathway to treat arginine-dependent cancers. Cancer Lett.

[5] Hanahan D, Weinberg RA (2011). Hallmarks of cancer: the next generation. Cell.

[6] Phillips MM, Sheaff MT, Szlosarek PW (2013). Targeting arginine-dependent cancers with arginine-degrading enzymes: opportunities and challenges. Cancer Res Treat.

[7] Dillon BJ, Prieto VG, Curley SA, Ensor CM, Holtsberg FW, Bomalaski JS, Clark MA (2004). Incidence and distribution of argininosuccinate synthetase deficiency in human cancers: a method for identifying cancers sensitive to arginine deprivation. Cancer.

[8] Kelly MP, Jungbluth AA, Wu BW, Bomalaski J, Old LJ, Ritter G (2012). Arginine deiminase PEG20 inhibits growth of small cell lung cancers lacking expression of argininosuccinate synthetase. Br J Cancer.

[9] Szlosarek PW, Klabatsa A, Pallaska A, Sheaff M, Smith P, Crook T, Grimshaw MJ, Steele JP, Rudd RM, Balkwill FR, Fennell DA (2006). *In vivo* loss of expression of argininosuccinate synthetase in malignant pleural mesothelioma is a biomarker for susceptibility to arginine depletion. Clin Cancer Res.

[10] Cheng PN, Lam TL, Lam WM, Tsui SM, Cheng AW, Lo WH, Leung YC (2007). Pegylated recombinant human arginase (rhArg-peg5,000mw) inhibits the *in vitro* and *in vivo* proliferation of human hepatocellular carcinoma through arginine depletion. Cancer Res.

[11] Ensor CM, Holtsberg FW, Bomalaski JS, Clark MA (2002). Pegylated arginine deiminase (ADI-SS PEG20,000 mw) inhibits human melanomas and hepatocellular carcinomas *in vitro* and *in vivo*. Cancer Res.

[12] Ascierto PA, Scala S, Castello G, Daponte A, Simeone E, Ottaiano A, Beneduce G, De Rosa V, Izzo F, Melucci MT, Ensor CM, Prestayko AW, Holtsberg FW (2005). Pegylated arginine deiminase treatment of patients with metastatic melanoma: results from phase I, II studies. J Clin Oncol.

[13] Glazer ES, Piccirillo M, Albino V, Di Giacomo R, Palaia R, Mastro AA, Beneduce G, Castello G, De Rosa V, Petrillo A, Ascierto PA, Curley SA, Izzo F (2010). Phase II study of pegylated arginine deiminase for nonresectable and metastatic hepatocellular carcinoma. J Clin Oncol.

[14] Izzo F, Marra P, Beneduce G, Castello G, Vallone P, De Rosa V, Cremona F, Ensor CM, Holtsberg FW, Bomalaski JS, Clark MA, Ng C, Curley SA (2004). Pegylated arginine deiminase treatment of patients with unresectable hepatocellular carcinoma: results from phase I/II studies. J Clin Oncol.

[15] Yau T, Cheng PN, Chan P, Chan W, Chen L, Yuen J, Pang R, Fan ST, Poon RT (2013). A phase 1 dose-escalating study of pegylated recombinant human arginase 1 (Peg-rhArg1) in patients with advanced hepatocellular carcinoma. Invest New Drugs.

[16] Qiu F, Chen YR, Liu X, Chu CY, Shen LJ, Xu J, Gaur S, Forman HJ, Zhang H, Zheng S, Yen Y, Huang J, Kung HJ, Ann DK (2014). Arginine starvation impairs mitochondrial respiratory function in ASS1-deficient breast cancer cells. Sci Signal.

[17] Rho JH, Qin S, Wang JY, Roehrl MH (2008). Proteomic expression analysis of surgical human colorectal cancer tissues: up-regulation of PSB7, PRDX1, and SRP9 and hypoxic adaptation in cancer. J Proteome Res.

[18] Vynnytska-Myronovska B, Bobak Y, Garbe Y, Dittfeld C, Stasyk O, Kunz-Schughart LA (2012). Single amino acid arginine starvation efficiently sensitizes cancer cells to canavanine treatment and irradiation. Int J Cancer.

[19] Vynnytska-Myronovska B, Kurlishchuk Y, Bobak Y, Dittfeld C, Kunz-Schughart LA, Stasyk O (2013). Three-dimensional environment renders cancer cells profoundly less susceptible to a single amino acid starvation. Amino Acids.

[20] Jang MH, Jun DY, Rue SW, Han K, Park W, Kim YH (2002). Arginine antimetabolite L-canavanine induces apoptotic cell death in human Jurkat T cells via caspase-3 activation regulated by Bcl-2 or Bcl-xL. Biochem Biophys Res Commun.

[21] Swaffar DS, Ang CY (1999). Growth inhibitory effect of L-canavanine against MIA PaCa-2 pancreatic cancer cells is not due to conversion to its toxic metabolite canaline. Anticancer Drugs.

[22] Worthen DR, Chien L, Tsuboi CP, Mu XY, Bartik MM, Crooks PA (1998). L-Canavanine modulates cellular growth, chemosensitivity and P-glycoprotein substrate accumulation in cultured human tumor cell lines. Cancer Lett.

[23] Vynnytska BO, Mayevska OM, Kurlishchuk YV, Bobak YP, Stasyk OV (2011). Canavanine augments proapoptotic effects of arginine deprivation in cultured human cancer cells. Anticancer Drugs.

[24] Huang HY, Wu WR, Wang YH, Wang JW, Fang FM, Tsai JW, Li SH, Hung HC, Yu SC, Lan J, Shiue YL, Hsing CH, Chen LT, Li CF (2013). ASS1 as a novel tumor suppressor gene in myxofibrosarcomas: aberrant loss via epigenetic DNA methylation confers aggressive phenotypes, negative prognostic impact, and therapeutic relevance. Clin Cancer Res.

[25] Nicholson LJ, Smith PR, Hiller L, Szlosarek PW, Kimberley C, Sehouli J, Koensgen D, Mustea A, Schmid P, Crook T (2009). Epigenetic silencing of argininosuccinate synthetase confers resistance to platinum-induced cell death but collateral sensitivity to arginine auxotrophy in ovarian cancer. Int J Cancer.

[26] Bobak Y, Kurlishchuk Y, Vynnytska-Myronovska B, Grydzuk O, Shuvayeva G, Redowicz MJ, Kunz-Schughart LA, Stasyk O (2016). Arginine deprivation induces endoplasmic reticulum stress in human solid cancer cells. Int J Biochem Cell Biol.

[27] Tan IK, Gajra B (2006). Plasma and urine amino acid profiles in a healthy adult population of Singapore. Ann Acad Med Singapore.

[28] Bence AK, Crooks PA (2003). The mechanism of L-canavanine cytotoxicity: arginyl tRNA synthetase as a novel target for anticancer drug discovery. J Enzyme Inhib Med Chem.

[29] Sato T, Stange DE, Ferrante M, Vries RG, Van Es JH, Van den Brink S, Van Houdt WJ, Pronk A, Van Gorp J, Siersema PD, Clevers H (2011). Long-term expansion of epithelial organoids from human colon, adenoma, adenocarcinoma, and Barrett's epithelium. Gastroenterology.

[30] Tsai WB, Aiba I, Lee S, Feun L, Savaraj N, Kuo MT (2009). Resistance to arginine deiminase treatment in melanoma cells is associated with induced argininosuccinate synthetase expression involving c-Myc/HIF-1α/Sp4. Mol Cancer Ther.

[31] Manca A, Sini MC, Izzo F, Ascierto PA, Tatangelo F, Botti G, Gentilcore G, Capone M, Mozzillo N, Rozzo C, Cossu A, Tanda F, Palmieri G (2011). Induction of arginosuccinate synthetase (ASS) expression affects the antiproliferative activity of arginine deiminase (ADI) in melanoma cells. Oncol Rep.

[32] Feun LG, Marini A, Walker G, Elgart G, Moffat F, Rodgers SE, Wu CJ, You M, Wangpaichitr M, Kuo MT, Sisson W, Jungbluth AA, Bomalaski J (2012). Negative argininosuccinate synthetase expression in melanoma tumours may predict clinical benefit from arginine-depleting therapy with pegylated arginine deiminase. Br J Cancer.

[33] Bobak YP, Vynnytska BO, Kurlishchuk YV, Sibirny AA, Stasyk OV (2010). Cancer cell sensitivity to arginine deprivation *in vitro* is not determined by endogenous levels of arginine metabolic enzymes. Cell Biol Int.

[34] Kobayashi E, Masuda M, Nakayama R, Ichikawa H, Satow R, Shitashige M, Honda K, Yamaguchi U, Shoji A, Tochigi N, Morioka H, Toyama Y, Hirohashi S (2010). Reduced argininosuccinate synthetase is a predictive biomarker for the development of pulmonary metastasis in patients with osteosarcoma. Mol Cancer Ther.

[35] Lan J, Tai HC, Lee SW, Chen TJ, Huang HY, Li CF (2014). Deficiency in expression and epigenetic DNA Methylation of ASS1 gene in nasopharyngeal carcinoma: negative prognostic impact and therapeutic relevance. Tumour Biol.

[36] Duldulao MP, Lee W, Le M, Chen Z, Li W, Wang J, Gao H, Li H, Kim J, Garcia-Aguilar J (2012). Gene expression variations in microsatellite stable and unstable colon cancer cells. J Surg Res.

[37] Ahmed D, Eide PW, Eilertsen IA, Danielsen SA, Eknaes M, Hektoen M, Lind GE, Lothe RA (2013). Epigenetic and genetic features of 24 colon cancer cell lines. Oncogenesis.

[38] Liang Y, Liu J, Feng Z (2013). The regulation of cellular metabolism by tumor suppressor p53. Cell Biosci.

[39] Hirschhaeuser F, Menne H, Dittfeld C, West J, Mueller-Klieser W, Kunz-Schughart LA (2010). Multicellular tumor spheroids: an underestimated tool is catching up again. J Biotechnol.

[40] Lovitt CJ, Shelper TB, Avery VM (2014). Advanced cell culture techniques for cancer drug discovery. Biology.

[41] D'Apolito O, Garofalo D, Gelzo M, Paris D, Melck D, Calemma R, Izzo F, Palmieri G, Castello G, Motta A, Corso G (2014). Basic amino acids and dimethylarginines targeted metabolomics discriminates primary hepatocarcinoma from hepatic colorectal metastases. Metabolomics.

[42] Lu Y, Wang W, Wang J, Yang C, Mao H, Fu X, Wu Y, Cai J, Han J, Xu Z, Zhuang Z, Liu Z, Hu H, Chen B (2013). Overexpression of arginine transporter CAT-1 is associated with accumulation of L-arginine and cell growth in human colorectal cancer tissue. PloS One.

[43] Crenn P, Vahedi K, Lavergne-Slove A, Cynober L, Matuchansky C, Messing B (2003). Plasma citrulline: A marker of enterocyte mass in villous atrophy-associated small bowel disease. Gastroenterology.

[44] Vynnytska-Myronovska BO, Kurlishchuk Y, Chen O, Bobak Y, Dittfeld C, Huther M, Kunz-Schughart LA, Stasyk OV (2016). Arginine starvation in colorectal carcinoma cells: Sensing, impact on translation control and cell cycle distribution. Exp Cell Res.

[45] D'Mello JPF (2012). Amino acid in human nutition and health.

[46] Thomas DA, Rosenthal GA (1987). Toxicity and pharmacokinetics of the nonprotein amino acid L-canavanine in the rat. Toxicol Appl Pharmacol.

[47] Zakalskiy AE, Zakalska OM, Rzhepetskyy YA, Potocka N, Stasyk OV, Horak D, Gonchar MV (2012). Overexpression of (His)_6_-tagged human arginase I in Saccharomyces cerevisiae and enzyme purification using metal affinity chromatography. Protein Expr Purif.

[48] Vovk OI, Igumentseva NI, Senchuk OY, Barska ML, Sybirna NO, Stasyk OV (2016). Effects of the combined arginase and canavanine treatment on leukemic cells *in vitro* and *in vivo*. Ukr Biochem J.

[49] Arundel CM, Nishioka K, Tofilon PJ (1988). Effects of alpha-difluoromethylornithine-induced polyamine depletion on the radiosensitivity of a human colon carcinoma cell line. Radiat Res.

[50] Policastro L, Duran H, Henry Y, Molinari B, Favaudon V (2007). Selective radiosensitization by nitric oxide in tumor cell lines. Cancer Lett.

[51] Gong H, Pottgen C, Stuben G, Havers W, Stuschke M, Schweigerer L (2003). Arginine deiminase and other antiangiogenic agents inhibit unfavorable neuroblastoma growth: potentiation by irradiation. Int J Cancer.

[52] Park H, Lee JB, Shim YJ, Shin YJ, Jeong SY, Oh J, Park GH, Lee KH, Min BH (2008). Arginine deiminase enhances MCF-7 cell radiosensitivity by inducing changes in the expression of cell cycle-related proteins. Mol Cells.

[53] Poland J, Sinha P, Siegert A, Schnolzer M, Korf U, Hauptmann S (2002). Comparison of protein expression profiles between monolayer and spheroid cell culture of HT-29 cells revealed fragmentation of CK18 in three-dimensional cell culture. Electrophoresis.

[54] Bowles TL, Kim R, Galante J, Parsons CM, Virudachalam S, Kung HJ, Bold RJ (2008). Pancreatic cancer cell lines deficient in argininosuccinate synthetase are sensitive to arginine deprivation by arginine deiminase. Int J Cancer.

[55] Marini JC, Didelija IC (2015). Arginine depletion by arginine deiminase does not affect whole protein metabolism or muscle fractional protein synthesis rate in mice. PloS One.

[56] Li L, Li Z, Chen D, Lu X, Feng X, Wright EC, Solberg NO, Dunaway-Mariano D, Mariano PS, Galkin A, Kulakova L, Herzberg O, Green-Church KB (2008). Inactivation of microbial arginine deiminases by L-canavanine. J Am Chem Soc.

[57] Friedrich J, Seidel C, Ebner R, Kunz-Schughart LA (2009). Spheroid-based drug screen: considerations and practical approach. Nat Protoc.

[58] Diermeier-Daucher S, Brockhoff G (2010). Dynamic proliferation assessment in flow cytometry. Curr Protoc Cell Biol.

[59] Begg AC, McNally NJ, Shrieve DC, Kärcher H (1985). A method to measure the duration of DNA synthesis and the potential doubling time from a single sample. Cytometry.

