# Supplementary material for: Co-application of canavanine and irradiation uncouples anticancer potential of arginine deprivation from citrulline availability
Source: Oncotarget. 2016 Sep 28;7(45):73292–308. doi: 10.18632/oncotarget.12320 (PMC5341980; doi:10.18632/oncotarget.12320)
Supplement: Supplementary file 1 [file oncotarget-07-73292-s001.pdf]

Co-application of canavanine and irradiation uncouples anticancer potential of arginine deprivation from citrulline availability

Supplementary Materials

Supplementary Table S1: Genetic profiles of human CRC cell lines

Cell line	ASS1 level	MSS/MSI	CIMP**	*TP53*	*KRAS**	*BRAF*	*APC*
NCI-H716	high	MSS[1]	–[1]	mut[2]	mut[1]	wt[1]	wt[1]
LS180	high	MSI[1]	–[1,3]	wt[1]	mut[7]	wt[3,7]	wt[1]
LS1034	high	MSS[4]	–[4]	mut[2,4]	mut[4]	wt[4]	mut[5]
LS513	high	MSS[6]	+[1]	wt[2,6]	mut[1]	wt[1]	wt[1]
SNU-C1	high	MSS[1]	–[1]	mut[1]	wt[1]	wt[1]	wt[1]
SW480	high	MSS[4]	–[4]	mut[4]	mut[4]	wt[4]	mut[1]
SW1417	high	MSS[1]	–[1]	mut[2]	wt[1,7]	mut[1,7]	mut[1]
Caco2	low	MSS[4]	+[4]	mut[4]	wt[4]	wt[4]	mut[1]
COLO 201	low	MSS[1]	+[1]	mut[1]	wt[1]	mut[1]	mut[1]
COLO 320HSR	low	MSS[7]	n.d.	mut[2]	wt[7]	wt[7]	mut[8]
HCT-116	low	MSI[1,4]	+[4]	wt[4]	mut[4]	wt[4]	wt[1]
LS411N	low	MSI[1]	+[1]	mut[2]	wt[1]	mut[1]	mut[1]
SW620	low	MSS[4]	+[4]	mut[4]	mut[4]	wt[4]	mut[1]
COLO 320DM	negative	MSS[1]	–[1]	mut[2]	wt[1]	wt[1]	mut[1]
HT29	negative	MSS[4]	+[1]	mut[4]	wt[4]	mut[4]	mut[1]
RKO	negative	MSI[4]	+[1,4]	wt[4]	wt[4]	mut[4]	wt[1]

CRC cell lines are categorized into two groups according to their ASS1 expression in 2-D monolayer culture under regular arginine-rich conditions. *TP53, KRAS, BRAF* and *APC* gene profiles (wt – wild type, mut – mutated) as well as microsatellite stable/instable (MSS/MSI) and CpG methylator phenotypes (CIMP) of the CRC cell lines have been summarized from literature data. Correlations between the ASS1 protein level (not detectable and low vs. high) and each genetic/epigenetic feature were calculated by the mean square contingency coefficient (phi coefficient) using SPSS 23 (IBM). *weak correlation ($p = 0.049$); **strong correlation ($p = 0.005$); n.d. - not defined.

Supplementary Table S2 : Statistical significance of SCD_{50} values

HT29 \ HCT-116	+ Arg (control)	+ Arg + Cav	– Arg	– Arg + Cav	– Arg + 0.05 mM Cit	– Arg + 0.05 mM Cit + Cav	– Arg + 0.4 mM Cit	– Arg + 0.4 mM Cit + Cav
+ Arg (control)		< 0.01	< 0.001	< 0.001	< 0.001	< 0.001	< 0.05	< 0.001
+ Arg + Cav	n.s			< 0.001		< 0.001		< 0.001
– Arg	< 0.001			< 0.001	< 0.001		< 0.001	
– Arg + Cav	< 0.001	< 0.001	< 0.001			n.s.		< 0.001
– Arg + 0.05 mM Cit	< 0.05		< 0.001			< 0.001	< 0.001	
– Arg + 0.05 mM Cit + Cav	< 0.001	< 0.001		< 0.001	< 0.001			< 0.001
– Arg + 0.4 mM Cit	n.s.		< 0.001		< 0.001			< 0.001
– Arg + 0.4 mM Cit + Cav	< 0.001	< 0.001		< 0.001		< 0.001	< 0.001	

+ Arg – medium with 0.40 mM arginine; –Arg – arginine-free medium; Cit – citrulline; Cav – 0.1 mM canavanine.

Significance levels (*p* values) for differences of SCD_{50} values from HCT-116 (red) and HT29 (black) spheroids, respectively, exposed to the various treatment conditions. The specific bootstrapping procedure and software applied for these statistical analyses are described in the Materials and Methods section.

Supplementary Table S3: Primary and secondary antibodies used in the study for Western blotting (WB) and immunofluorescent staining in frozen tissue sections (IF/F)

Primary antibody	Manufacturer	Catalog #	Application	Dilution
anti-ASS1, mouse monoclonal	Acris Antibodies	AM06726PU-N	WB	1:1000
anti-cleaved-PARP, rabbit polyclonal	Cell Signaling Technology	9541	WB	1:1000
anti-phospho-RB, rabbit polyclonal	Cell Signaling Technology	9308	WB	1:1000
anti-GAPDH rabbit polyclonal	Santa Cruz Biotechnology	sc-25778	WB	1:1000
anti-β-ACTIN, mouse monoclonal	Abcam	ab6276	WB	1:10000
anti-α-TUBULIN, mouse monoclonal	Merck Millipore	05-829	WB	1:10000
anti-ASS1, mouse monoclonal	Abcam	ab124465	IF/F	1:200
isotype control, mouse IgG1	Abcam	ab91535	IF/F	1:1000
**Secondary antibody**				
HRP-conjugated, polyclonal swine anti-rabbit	Dako	P 0399	WB	1:5000
HRP-conjugated, polyclonal rabbit anti-mouse	Dako	P 0260	WB	1:5000
Alexa^{594}-conjugated goat anti-mouse	Invitrogen	A11032	IF/F	1:500

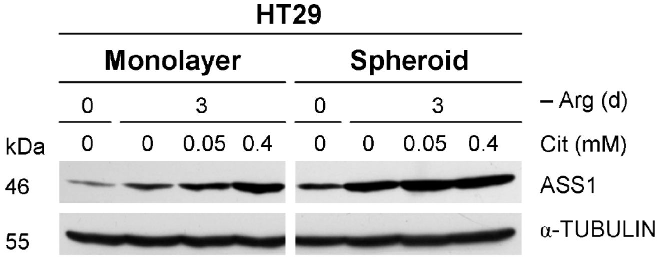

Supplementary Figure S1: ASS1 expression in HT29 cells. Representative Western blot of protein extracts from monolayer and spheroid cultures of HT29 cells harvested directly before (day 0) or after a 3-day exposure to arginine-free (–Arg) medium alone, or supplemented with the indicated concentrations of citrulline (Cit). Total protein extracts were probed with an anti-ASS1 antibody. α-TUBULIN was used as loading control.

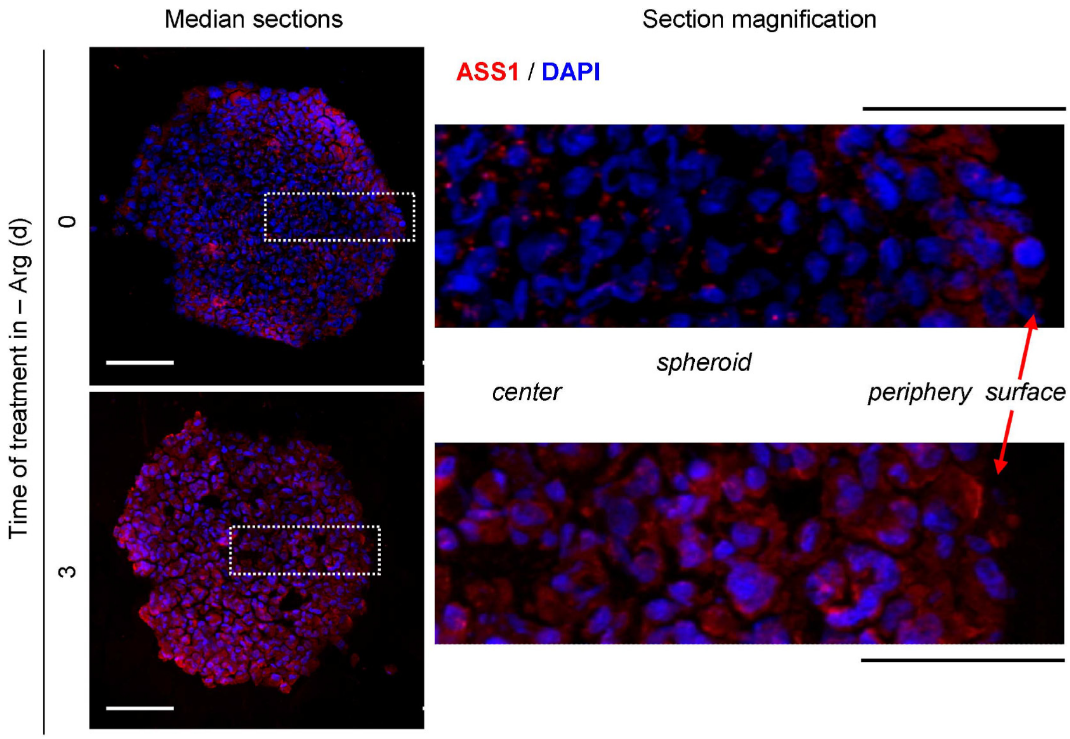

Supplementary Figure S2: Distribution of ASS1 protein in an HCT-116 spheroid. Representative fluorescence images of 10 µm median sections from frozen HCT-116 spheroids directly before (day 0) or after 3 days of incubation in arginine-free medium (– Arg) are shown. Immunodetection was performed with an anti-ASS antibody followed by an Alexa[594]-conjugated secondary antibody and DAPI counterstaining. Scale bar = 100 µm.

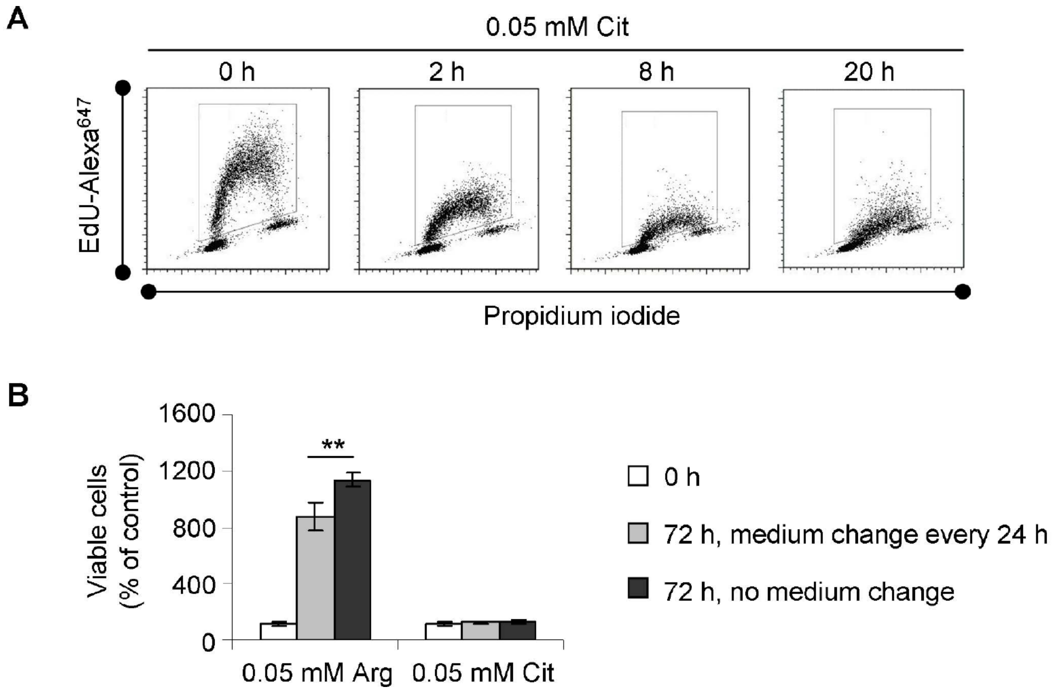

Supplementary Figure S3: Physiological citrulline is insufficient to support cell cycling upon arginine deprivation. (**A**) Representative flow cytometric dot blot diagrams of EdU-labeled monolayer HT29 cells exposed to arginine-free medium with 0.05 mM citrulline (0.05 mM Cit) for 0, 2, 8, or 20 h are shown. (**B**) Viability of monolayer HT29 cells was determined before (0 h) or after exposure to arginine-free medium supplemented with the indicated concentrations of arginine (0.05 mM Arg) or citrulline (0.05 mM Cit) for 72 h. Cells were either incubated for defined time intervals without medium renewal, or the specific media were refreshed on a daily basis.

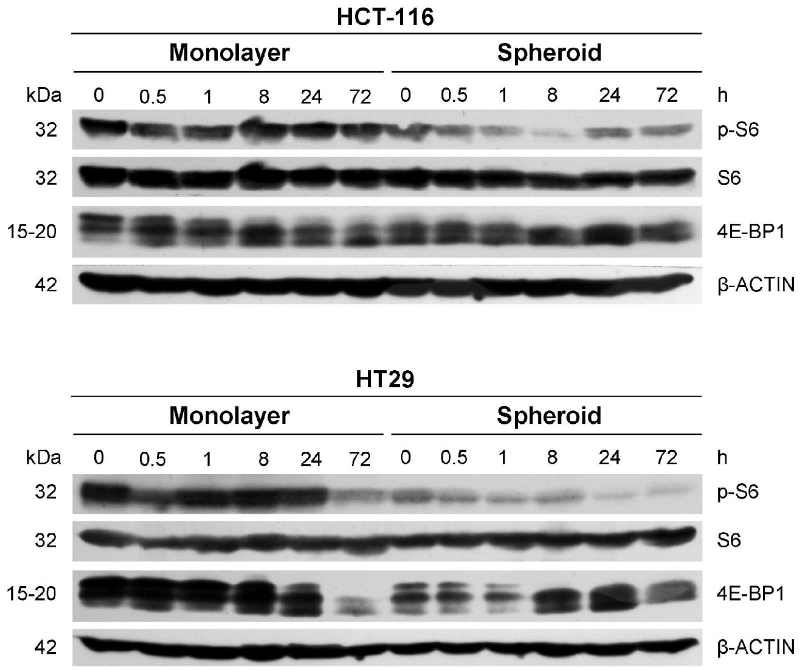

Supplementary Figure S4: mTOR signaling in CRC cells exposed to combination of arginine deprivation and canavanine treatment. Representative Western blots of protein extracts from monolayer and spheroid cultures of HCT-116 and HT29 cells harvested directly before (0 h) or after exposure to arginine-free medium supplemented with 0.1 mM canavanine for the indicated periods of time are shown. Total protein extracts were probed with antibodies against downstream mTORC1 targets (S6 and 4E-BP1). β-ACTIN was used as loading control.

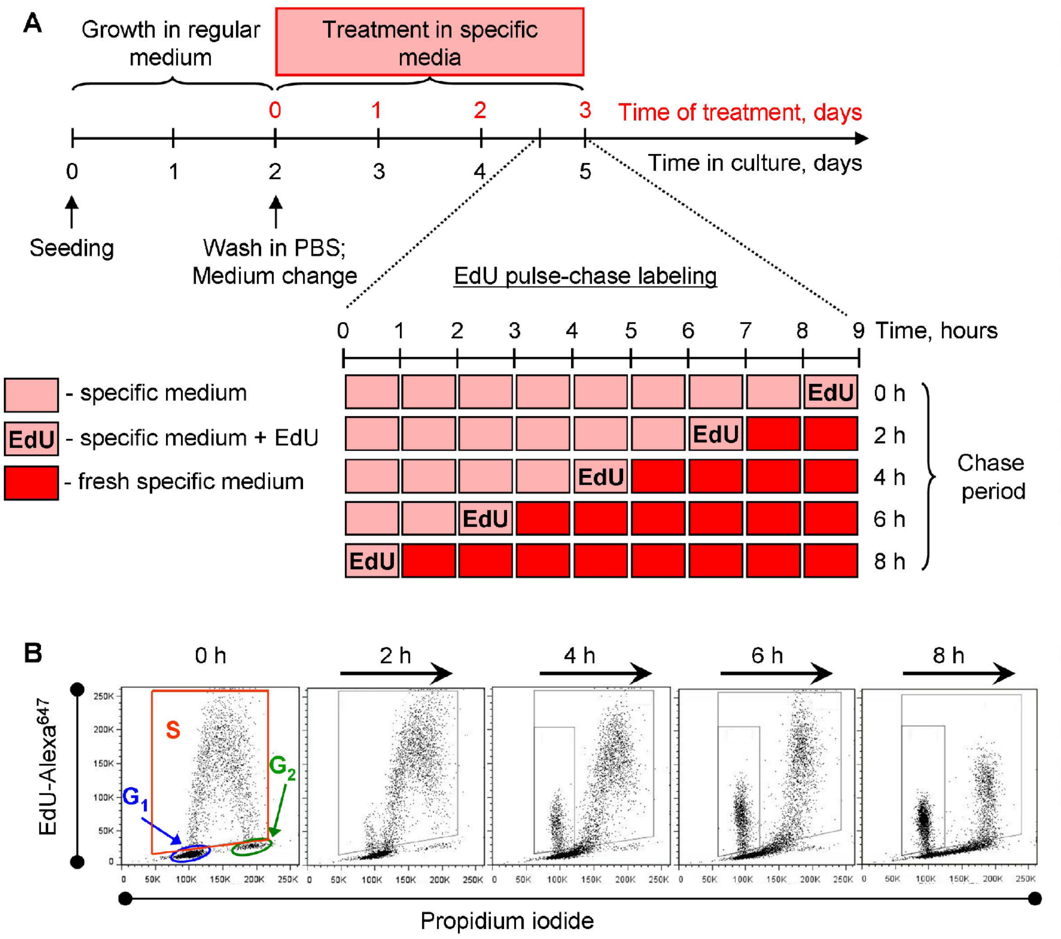

Supplementary Figure S5: EdU incorporation experimental setup for monolayer HT29 cells. (**A**) Scheme of the EdU pulse and chase periods scheduled within 72 h of treatment in specific medium is shown. (**B**) Representative dot blot diagrams of HT29 cells exposed to control conditions (0.40 mM arginine) for a total of 72 h including chase periods of 0, 2, 4, 6 or 8 h allowed tracing the movement of EdU-labeled cells through the cell cycle. The G_1, S and G_2/M cell populations are delineated in the first dot pot diagram at the time point directly after EdU labeling.

REFERENCES

1. Mouradov D, Sloggett C, Jorissen RN, Love CG, Li S, Burgess AW, Arango D, Strausberg RL, Buchanan D, Wormald S, O'Connor L, Wilding JL, Bicknell D, et al. Colorectal cancer cell lines are representative models of the main molecular subtypes of primary cancer. Cancer Res. 2014; 74:3238–3247.
2. Liu Y, Bodmer WF. Analysis of P53 mutations and their expression in 56 colorectal cancer cell lines. Proc Natl Acad Sci USA. 2006; 103:976–981.
3. Dawson H, Galvan JA, Helbling M, Muller DE, Karamitopoulou E, Koelzer VH, Economou M, Hammer C, Lugli A, Zlobec I. Possible role of Cdx2 in the serrated pathway of colorectal cancer characterized by BRAF mutation, high-level CpG Island methylator phenotype and mismatch repair-deficiency. Int J Cancer. 2014; 134: 2342–2351.
4. Ahmed D, Eide PW, Eilertsen IA, Danielsen SA, Eknaes M, Hektoen M, Lind GE and Lothe RA. Epigenetic and genetic features of 24 colon cancer cell lines. Oncogenesis. 2013; 2:e71.
5. Rowan AJ, Lamlum H, Ilyas M, Wheeler J, Straub J, Papadopoulou A, Bicknell D, Bodmer WF, Tomlinson IP. APC mutations in sporadic colorectal tumors: A mutational "hotspot" and interdependence of the "two hits". Proc Natl Acad Sci USA. 2000; 97:3352–3357.
6. Gayet J, Zhou XP, Duval A, Rolland S, Hoang JM, Cottu P, Hamelin R. Extensive characterization of genetic alterations in a series of human colorectal cancer cell lines. Oncogene. 2001; 20:5025–5032.
7. Medico E, Russo M, Picco G, Cancelliere C, Valtorta E, Corti G, Buscarino M, Isella C, Lamba S, Martinoglio B, Veronese S, Siena S, Sartore-Bianchi A, Beccuti M, Mottolese M, Linnebacher M, et al. The molecular landscape of colorectal cancer cell lines unveils clinically actionable kinase targets. Nat Commun. 2015; 6:7002.
8. Yeh JJ, Routh ED, Rubinas T, Peacock J, Martin TD, Shen XJ, Sandler RS, Kim HJ, Keku TO and Der CJ. KRAS/BRAF mutation status and ERK1/2 activation as biomarkers for MEK1/2 inhibitor therapy in colorectal cancer. Mol Cancer Ther. 2009; 8:834–843.
